# A tale of five stories: Defence spending and economic growth in NATO´s countries

**DOI:** 10.1371/journal.pone.0245260

**Published:** 2021-01-11

**Authors:** Paula Gómez-Trueba Santamaría, Alfredo Arahuetes García, Tomás Curto González

**Affiliations:** 1 Programme in Corporate and Territorial Competitiveness, Innovation and Sustainability (CETIS) at Comillas Pontifical University, ICAI-ICADE, Madrid, Spain; 2 Department of Economy, Faculty of Economics and Business Administration at Comillas Pontifical University, ICAI-ICADE, Madrid, Spain; 3 Department of Quantitative Methods, Faculty of Economics and Business Administration at Comillas Pontifical University, ICAI-ICADE, Madrid, Spain; 4 Economy Department, Faculty of Economics and Business Administration at Universidad CEU San Pablo, Madrid, Spain; Universidade da Coruna, SPAIN

## Abstract

This article examines the relationship between defence expenditure and its impact on the growth of NATO’s countries between 2005 and 2018. The aim is to determine if this relation exists and to test if it is possible to discover different models across the countries. The results obtained using the Arellano–Bond estimator, suggest that there is more than one model, and confirm, through the poolability test, the existence of five different groups of countries within the Alliance, with different impacts of the defence expenditure on their gross domestic product. These findings are in line with the review of existing literature that reveals heterogeneity in the results due to different parameters used.

## 1. Introduction

**Fig 1:**
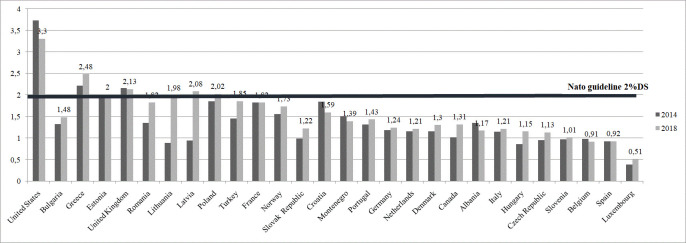
Military expenditure as a share of GDP. Source: Self-elaboration according to NATO data.

In the US 2016 electoral process, President Donald Trump already affirmed that the NATO was an obsolete institution, even considering it a relic of the Cold War, noting that “we´re dealing with NATO from days of the Soviet Union, which no longer exist (…)” [1]. He has called for the need for changes in NATO's fields of action, such as terrorism, without acknowledging the Alliance's support for the US military in the Middle East. But, above all, he has made the defence spending the main point of his claims to the NATO countries, pointing out that the existence of an asymmetry between the US military spending and that of the rest of the NATO members, threatening to withdraw troops and funds given that the lack of commitment of the Allies in terms of funding and modernization. After the end of the Cold War, the United States of America reduced their military presence in Europe. However, the increase in the scale of tension, in different fronts, with Russia determined that, in recent years, the USA increased again their military presence in Europe with more troops and equipment, more investment in infrastructure and more exercises. For their part, the European Allies countries are more convinced than ever of the importance of their commitment to NATO, and after years of reducing defence spending, all of them and Canada are now investing significantly more in defence. However, he maintains his insistence that countries must increase their military spending up to the committed level of 2% of GDP [2] (see Fig 1). He has even gone so far as to point out that there are countries, such as Germany, that should pay to the US for the defence that it provides to them directly and within NATO.

For some circumstances or others, there is no doubt that the issue of defence spending is on the table in Europe, with important economic implications over our societies. For all these reasons, it has been consider that it would be relevant, for our societies and policymakers, to carry out a study that would shed some light on the relationship between defence spending and economic growth in NATO countries.

In these countries, defence spending depends as much on economic factors [3] as on political and sociological factors. Implementing a defence policy that guarantees the necessary funding of the armed forces proves difficult if social awareness about the relevance of the armed forces has not been raised, for example to guarantee the defence of the national territory and to ensure the fulfilment of international commitments, such as those entailed by the NATO membership.

This is the reason why the positive results in the GDP by the military expenditure, obtained in this paper, intend to be an empirical contribution to the debate, on the effects of the relationship between defence spending and GDP for both the NATO countries as a whole, and for the five groups of countries that would make up the Alliance.

This paper is not exactly an extension of some existing work such us Odehnal [3, 4], Ozun [5] or Spangler [6], among others. None of them analyse the relationship between the military expenditure and the economic growth with a dynamic panel data model. In fact, there are no many articles about NATO. Its novelty is to subcategorize NATO’s countries into five categories according to their historical and economic characteristics. Moreover, the use of a dynamic model is a novelty itself due to there is few papers that use this technique. Finally, the adoption of the poolability test to our case has allowed confirming the existence of five different models in a panel of twenty eight NATO countries, rarely analysed in the literature so far.

To meet these objectives, this article is structured in five sections, in addition to this introduction. In the second section, the literature review is carried out, in the third section, the data and methodology are presented, in the fourth section the econometric study is developed using an Arellano-Bond dynamic panel analysis. The results obtained are presented in the fifth section. Finally, the conclusions and implications are presented in the sixth section.

## 2. Conceptual framework

Since Benoit [7, 8] a broad branch of literature has been focused on the empirical analysis of the relationship between military spending and economic growth. This empirical literature has grown exponentially, over the years, trying to understand the link between defence spending and economic growth for many countries. In very broad terms, it can be said that there is no consensus on the impact that military expenditure can have on the economy of a region [9–13]. The main reason for this heterogeneity is the differences in the samples analysed in terms of approach, methodology, countries, variables, and time, among others. However, these studies, carried out from the perspective of the defence economy, have found, in many cases, positive effects between defence spending and economic growth.

In this context, the following section is going to be divided into four subsections, to review the existing literature by summarizing in terms of approach, method of analysis, countries and other variables.

### 2.1 Approach

Regarding the approach of the study, there are articles that, from the point of view of the demand, analysed the effect of defence expenditure based on Keynesian theory and the multiplier effect. These studies agreed that an increase in this type of expenditure would increase aggregate demand and, consequently, would generate an increase in the use of productive capital and, therefore, employment. We can cite, among others, Benoit [8], Smith [14] and Faini et al. [15].

There are studies that analysed the effects from the point of view of the supply, obtaining very different results. Some studies have confirmed that investment in defence implied the lack of allocation of resources in other productive sectors of the economy, and that, therefore, they reduced productivity in the long term [16]. On the contrary, other authors studied the positive impact that defence expenditure generated on other sectors of the economy thanks to technological and human development [17]. Finally, it is worth mentioning those studies that integrated both effects, among which we can mention those carried out by Deger et al. [9, 18], Antonakis [19] and Galvin [20].

### 2.2 Method of analysis

Based on the perspective from which it is investigated, we could speak, on the one hand, of single-equation studies, based on neoclassical or Keynesian formulations, depending on whether the analysis is performed from supply or demand. On the other hand, we have other multiple-equation models, which try to analyse the influence of the two effects. The main difference is that multiple-equation models do not ignore the determinants of growth and assume defence expenditure as an exogenous variable. They also allow simultaneous analysis of the positive effects of defence expenditure on aggregate demand through spin-offs and spillovers, together with the indirect negative effects derived from crowding out.

In the first group, on the demand side, the studies of Benoit [8], Smith [14] and Faini et al. [15] are noteworthy. From the supply side, applying Feder's [21] model and establishing a positive relationship between the variables, the studies of Atesoglu et al. [22], Ram [23] and Ward et al. [24] should be noted. Other authors found no relationship under this methodology, such as Biswas et al. [25], Alexander [26], Huang et al. [27, 28], Adams et al. [29] and Nikolaidou [30].

In addition, other authors established a negative relationship using the Barro, Solow and Augmented Solow models, such as Aizenman et al. [31], Yakovlev [17], Dunne et al. [10, 32], Hou et al. [33–35].

In the group of multiple-equation models, the studies of Deger et al. [9, 18, 36], Deger [37, 38], Scheetz [39], Antonakis [19], Sezgin [40] and Galvin [20], using the Deger methodology that combines the effects of demand and supply, concluded on the domain of negative effects.

Finally, it is worth mentioning the use of two specific methodologies: the Granger methodology (extensively used) and the panel estimation through FEM (Fixed Effect Models), GMM (Generalized Method of Moments), and FGLS (Feasible generalized least squares). In the first group, we can distinguish, among others, the studies of Chowdhury [41], Seiglie et al. [42], Pradhan [43], Balan [44] and Su et al. [45], while in the second group we can highlight the studies of Yildirim et al. [46], Hou et al. [33, 35], Dunne et al. [47] and Spangler [6].

### 2.3 Countries

Regarding the countries under study, those studies carried out on OECD member countries should be mentioned. Smith [14], using demand-side models, analysed between 1954 and 1973, fourteen OECD member countries, concluding that defence expenditure reduced investments. On the other hand, Cappelen et al. [48], through a model of three equations for the study of seventeen OECD countries in the period 1960–1981, reached the same conclusion as the previous author.

Landau [49] conducted a study on seventeen prosperous OECD countries during the period of 1950–1990, determining that for low levels of military expenditure, an increase in said military budget would boost the growth of the economy, but only up to a certain level, after which the impact would change and would negatively affect growth.

Notwithstanding the foregoing, Lee et al. [50] showed a positive relationship in a study conducted on twenty-seven OECD member countries in the period 1988–2003, using unit root tests.

Finally, it is worth highlighting the study conducted by Hou et al. [35] on twenty-one OECD member countries in the period 1960–2009, in which they used the Augmented Solow methodology and other panel data (fixed effect models—FEM, generalized method of moments—GMM and feasible generalized least squares—FGLS), and who observed a negative effect, of a minimal nature, on the growth of the economy.

The analysis of the relationship between defence expenditure and the growth of the economy in the so-called less developed countries (hereinafter, LDC) has also been the subject of numerous studies. It is important to note the studies of Benoit [8], carried out on forty-four countries with a positive result in the relationship under study; Deger [37], who analysed this correspondence in fifty LDC through a multiple-equation system that demonstrated a two-way relationship between defence expenditure and growth in the economy; Joerding [51], who found no evidence in the analysis conducted on fifty-seven LDC between 1962 and 1977; Chowdhury [41], who analysed fifty-five countries in the same environment through Granger's causality methodology, and confirmed not only the bidirectionality of the relationship between the variables, but also the impact that the growth of the economy had on the budget that was assigned to defence in certain countries.

Dakurah et al. [52] analysed the effect in sixty-nine LDC using the Granger test and concluded their study by confirming the absence of this relationship in most of them. On the other hand, Galvin [20], who conducted a study on sixty-four countries using a multiple-equation model of both effects, supply and demand, concluded there was a negative effect. D’ Agostino et al. [53], in a study on one hundred and nine LDC, concluded that there was a negative effect of defence expenditure on economic growth.

Another group of countries under study are those that are grouped under the general denomination of Middle East, and here we must highlight the studies of Lebovic et al. [54], conducted on twenty countries with a negative association result; Yildirim et al. [46], with a positive result; Pan et al. [55], who focused their research study on ten countries in the period 1988–2010, using Granger's causality test, and found that the relationship went from economic growth to defence expenditure in certain countries. In this area, it is worth mentioning Balan’s [44] analysis carried out on twelve countries in the period between 1988 and 2013, using the Granger methodology, which resulted in a positive effect between the variables analysed. Finally, Coutts et al. [56] analysed eighteen countries grouped under the acronym of MENA.

There are also numerous studies carried out on countries of the European Union, such as the studies by Kollias et al. [57, 58] who, using the dynamic panel methodology with fixed effects, affirmed the existence of a positive relationship between defence expenditure and the growth of the economy. Other examples are the studies performed by Mylonidis [59], Chang et al. [60], who applied the Granger methodology, Hunter [61], Michael et al. [62], Daddi et al. [63], Dimitraki et al. [64], Aben et al. [65] and Berg et al. [66], among other studies.

However, there are few studies carried out on other European countries in relation with the NATO. It is interesting to highlight, among others, Odehnal et al [3, 4] Ozun [5], or Spangler [6].

### 2.4 Other variables

At this point, it is necessary to mention the studies that related not only the growth and defence variables, but also those that incorporated additional variables to the model. Thus, two authors incorporated the variable "political instability". The first one, Blomberg [67] concluded that in countries of Africa and Latin America, the increase in political instability inhibited economic growth, and defence expenditure reduced political instability and, insignificantly, the growth of the economy. On the other hand, Balan [44] analysed twelve countries of the Middle East, for the period between 1988 and 2013, with Granger’s causality methodology, and determined the clearly positive effect between the different variables.

Pradhan [43], in a study on China, India, Nepal and Pakistan in the period 1988–2003, introduced the variable "public debt", determining, through the Granger methodology, that there was a bidirectional relationship between public expenditure and the growth of the economy in the cases of China and India; a one-way relationship of defence expenditure on the growth of the economy in China and Nepal; and a one-way relationship of public debt towards defence expenditure in India.

In 2012, D’Agostino et al. [68], using the panel methodology for the analysis of fifty-three African countries in the period between 2003 and 2007, determined that reduced levels of economic growth were related to high levels of defence expenditure and “corruption". These same variables were analysed by Ali et al. [69] in a total of fifty-nine countries, concluding that there was a direct relationship between defence expenditure and "corruption".

Also using panel methodology, Chang et al. [60], in 2015, analysed how the variables of defence, economic growth and investment in fifteen countries of the European Union were related during the period 1988–2010. The result was the existence of a link between the growth of the economy and defence expenditure, as well as between defence expenditure and investment.

Other variables have also been analysed together with the military expenditure. Solarin [70] analysed how the defence expenditure could be related to the globalization. Xu et al. [71] examined if the defence expenditure could be inflationary. The debate of Guns versus Butter is still on the table. Coutts et al. [56] and Fan et al [72] evaluated the defence expenditure in relation with health expenditure. Kishore et al. [73] studied how impact the military expenditure in the human development index. Other authors, such as, Dunne et al. [47], Aben et al. [65], Aziz et al. [74], Bolzan et al. [75], and Ahmed et al. [76], have investigated the relationship between defence expenditure with external debt, state power, foreign direct investment flows, defence imports and energy consumption, among others. In summary, all these studies highlight that it is important to note that the military expenditure has influence in other productive activities due to the relation that exists between defence expenditure and other factors.

In view of the very varied results obtained in the mentioned literature (see Table 1), and the analysis of the academic discussion that exist, this article aims to contribute to the literature through the study of the impact of defence expenditure on the growth of the NATO’s countries between 2005 and 2018, in order to determine what type of causality exists between both variables.

**Table 1:** Literature review summary.

		Author	Bibliography number			Author	Bibliography number
**Approach**	**Keynesian theory**	Benoit	8	**Countries**	**OECD**	Smith	14
		Smith	14			Cappelen et al.	48
		Faini et al.	15			Landau	49
	**Supply side**	Chan	16			Lee et al.	50
		Yakovlev	17			Hou et al.	35
	**Both**	Deger et al.	9		**LDC**	Benoit	8
		Deger et al.	18			Deger	37
		Antonakis	19			Joerding	51
		Galvin	20			Chowdhury	41
**Method of Analysis**	**Demand side**	Benoit	8			Dakurah et al.	52
		Smith	14			Galvin	20
		Faini et al.	15			D'Agostino et al.	53
	**Supply side**	Atesoglu et al.	22		**Middle East**	Lebovic et al.	54
		Ram	23			Yildirim et al.	46
		Ward et al.	24			Pan et al.	55
		Biswas et al.	25			Balan	44
		Alexander	26			Coutts et al.	56
		Huang et al.	27		**European Union**	Kollias et al.	57
		Huang et al.	28			Kollias et al.	58
		Adams et al.	29			Mylonidis	59
		Nikolaidou	30			Chang et al.	60
	**Uniequation model**	Aizenman et al.	31			Hunter	61
		Yakovlev	17			Michael et al.	62
		Dunne et al.	10			Daddi et al.	63
		Dunne et al.	32			Dimitraki et al.	64
		Hou et al.	33			Aben et al.	65
		Hou et al.	34			Berg et al.	66
		Hou et al.	35		**NATO**	Odehnal et al.	3
	**Multiple equation model**	Deger et al.	9			Ozun	5
		Deger et al.	18			Spangler	6
		Deger et al.	36	**Variables**	**Political instability**	Blomberg	67
		Deger	37			Balan	44
		Deger	38		**Public Debt**	Pradhan	43
		Scheetz	39		**Corruption**	D'Agostino et. al	68
		Antonakis	19			Ali et al	69
		Sezgin	40		**Investment**	Chang et al.	60
		Galvin	20		**Globalization**	Solarin	70
	**Granger**	Chowdhury	41		**Inflation**	Xu et al.	71
		Seiglie et al.	42		**Health expenditure**	Coutts et al.	56
		Pradhan	43			Fan et al.	72
		Balan	44		**HDI**	Kishore et al.	73
		Su et al.	45		**External debt**	Dunne et al.	47
	**Estimation Panel**	Yildirim et al.	46		**State power**	Aben et al.	65
		Hou et al.	35		**FDI**	Aziz et al.	74
		Dunne et al.	47		**Defence imports**	Bolzan et al.	75
		Spangler	6		**Energy consumtion**	Ahmed et al.	76

Source: Self-elaboration

## 3. Data and methodology

This study is part of the small number of studies focused on the interaction between military spending and economic growth in the twenty eight NATO countries, compared to those conducted for developing countries.

It is an empirical study carried out from the Keynesian perspective of defence economics and its relationship with economic growth, and not from the neoclassical literature on economic growth towards defence economics. To some extent, this investigation is in line with the articles about NATO [3–6] in which, the results, after using multiples variables, confirm, above all, the relation between the military expenditure and the economic growth.

**Fig 2:**
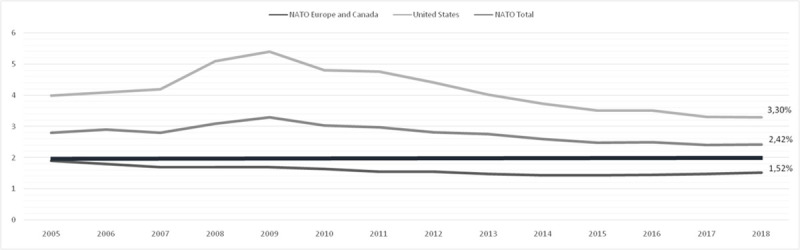
Military expenditure as a share of GDP from 2005 to 2018. Source: Self-elaboration according to NATO data.

Thus, this paper determines empirically not only the relationship of causality between defence expenditure (MilExp in current USD) and economic growth (GDP in current USD), in NATO countries, through a panel data model using the Arellano-Bond estimator, for the period between 2005 and 2018, using data from the World Bank transformed into logarithms (LnMilExp and LnGDP) (see Fig 2), and the software package used is STATA 16, but also it contributes to the identification and confirmation of five sub models inside of the Alliance. According to Judson et al. [77] “Use of panel data in estimating common relationships across countries is particularly appropriate because it allows the identification of country-specific effects that control for missing or unobserved variables”.

Moreover, even though most of the studies have not analyzed the stationarity, because of using time series, this paper has carried out this analysis to determine if the relationship between the variables was not spurious, ensuring the validity of the data and their stability in the long run. Therefore, the Levin–Lin–Chu Unit Root Test has been carried out. If a variable does not pass the test, it has been transformed taking first differences.

Therefore, the causality of the relation between military expenditure and economic growth has been examined once the data has been transformed into logarithms in order to reduce deviations in case of atypical observations (LnMilExp and LnGDP). Several models could be used in a dynamic data panel, highlighting among others, the fixed effect models (FEM), the feasible generalized least squares (FGLS), the random effects estimator (RE), and the generalized method of moments (GMM). Taking into consideration the panel’s characteristics, the lagged variables and the regression equations that would be estimated, the proposed method would be to introduce dynamics to panel data, using Arellano Bond throughout GMM, with a threshold for significance of 5%.

According with Nickell [78] and Dunne et al. [79], the results obtained by modelling a lagged dependent variable with a fixed effects estimator, could be biased and inconsistent. Regarding the random effect’s estimator, Dunne et al. [79] highlights that “it has the disadvantage that it is rendered inconsistent by correlation between the fixed effects and the repressors”. To achieve a consistent and efficient model, Dunne et al. [79] and Spangler [6] proposed the Arellano and Bond [80] dynamic panel method.

Finally, once the model is estimated, the next question to answer is to determine if there is just a unique panel data model for all the NATO countries or if different models could be distinguished according to some characteristics of the countries. Odehnal et al. [3, 4] analysed the NATO countries divided into two groups: the traditional and the ‘new members’ countries. However, to avoid the heterogeneity if the parameters differ among groups [81], this paper contributes to previous research by dividing the Alliance into five groups according to the historical and economical characteristics after the WWII until today. Their existence has been confirmed through the poolability test.

The resulting groups are: Countries which were defeated or neutral during the Second World War (Germany, Italy, Spain and Portugal); countries which developed nuclear weapons during the early years of the Cold War (USA, UK, and France); the Easter European countries formerly linked to the old Soviet Union (Albania, Bulgaria, Czech Republic, Estonia, Latvia, Lithuania, Romania, Slovakia, Slovenia, Croatia, Montenegro, Poland and Hungary); countries who were in constant conflict (Greece and Turkey) and the rest of the allies (Belgium, Canada, Denmark, Netherlands, Luxembourg and Norway).

The distribution of military spending in each of these five groups is very different among the four main categories (equipment, personnel, infrastructure and others). In this respect, it is important to highlight that the three countries with nuclear arsenals are the ones with the lowest personnel participation in the distribution of defence expenditure, and the ones with more spending on equipment (which includes equipment and R&D to devoted to major equipment) and other expenses (which includes operations, maintenance, R&D and other expenditures not allocated among the others categories) (see Table 2). The pattern of the countries that spend the most on personnel is not very clear. In this group are some countries belonging to the group linked to the former USSR, but not the Baltic countries or Hungary that spends more on equipment and other expenses. However, personnel expenditure is very high in Belgium, Italy, Portugal, and Spain as well. In all of them the weight of equipment and other expenses is quite different. For its part, in the group of countries in conflict, the personnel expenditure is very high in Greece but not in Turkey, which spends more specially on equipment (see Table 2).

**Table 2:** Distribution of defence expenditure of the NATO’s countries (by main categories and percentage of the total).

			NATO Countries									
		Distribution of Defence Expenditure (by main categories, % total)										
		Equipment			Personnel			Infrastructure			Others	
	2006	2013	2018	2006	2013	2018	2006	2013	2018	2006	2013	2018
Albania	-	16,29	9,42	-	75,25	70,70	-	1,17	1,09	-	7,30	18,79
Belgium	5,9	2,84	10,15	75,30	77,34	70,69	2,00	2,28	1,43	16,80	17,53	17,72
Bulgaria	14,1	4,52	9,65	51,80	65,37	62,99	0,60	0,47	2,62	33,50	29,64	24,74
Canada	11,8	11,16	11,94	46,60	52,44	51,02	2,60	4,12	3,58	39,00	32,28	33,46
Croatia	-	10,72	3,37	-	68,06	76,96	-	1,21	1,00	-	20,01	18,67
Czech Republic	14,6	9,49	11,16	47,40	62,03	54,57	8,30	2,72	5,31	29,70	25,75	28,95
Denmark	15,4	11,26	11,66	48,50	51,74	49,88	4,10	1,16	1,49	32,00	35,84	36,97
Estonia	14,5	14,48	16,73	26,00	39,83	34,27	16,40	11,54	8,63	43,20	34,14	40,36
France	22,8	28,56	23,66	57,40	49,23	46,90	3,70	2,30	3,51	16,20	19,91	25,92
Germany	15,0	12,74	12,36	57,10	49,86	47,99	3,60	3,55	4,15	24,30	33,84	35,49
Greece	14,9	12,06	11,03	73,80	74,56	78,76	1,00	0,63	0,62	10,20	12,75	9,60
Hungary	9,0	11,08	20,35	51,20	48,96	39,98	8,10	2,32	4,85	31,70	37,64	34,82
Italy	7,2	12,51	21,12	81,90	75,00	65,66	0,60	1,57	1,92	10,30	10,93	11,30
Latvia	12,3	12,09	31,19	39,20	52,98	34,21	9,70	6,26	6,97	38,80	28,68	27,63
Lithuania	17,0	9,23	36,98	54,80	66,53	37,47	3,50	2,04	2,24	24,60	22,20	23,30
Luxembourg	8,7	14,57	45,18	76,50	51,10	33,42	2,00	11,81	5,05	12,80	22,52	16,35
Montenegro	-	1,32	11,05	-	87,68	72,87	-	0,09	2,24	-	10,91	13,84
Netherlands	16,8	12,57	16,39	47,80	58,53	51,16	3,50	2,74	3,46	31,90	26,16	28,99
Norway	19,4	18,89	25,60	45,40	42,21	36,43	4,30	5,33	6,67	30,90	33,88	31,30
Poland	18,2	13,90	27,51	53,80	57,70	46,14	3,80	5,62	3,45	24,20	22,78	22,89
Portugal	8,9	8,65	9,78	76,20	79,85	74,84	1,80	0,04	0,12	13,10	11,46	15,26
Romania	24,0	10,71	33,48	59,80	78,99	54,48	2,10	1,16	1,54	14,20	9,13	10,50
Slovak Republic	12,7	7,39	22,27	49,10	70,14	54,74	5,20	0,29	2,00	33,00	22,19	20,99
Slovenia	12,2	1,27	5,86	60,10	80,52	72,38	0,80	1,33	1,40	26,90	16,88	20,36
Spain	21,7	12,37	21,83	53,50	68,25	59,64	2,80	0,67	0,64	22,00	18,71	17,89
Turkey	34,4	26,89	37,64	48,40	54,58	45,18	2,40	2,72	2,53	14,80	15,80	14,65
United Kingdom	21,4	21,89	22,19	40,40	37,85	33,82	2,60	2,04	2,99	35,60	38,22	41,00
United States	23,8	25,83	27,06	36,90	34,38	39,28	1,10	2,08	1,17	38,10	37,72	32,49

Source: Figures prepared from NATO, www.nato.int

Equipment: includes major equipment expenditure and R&D to devote to major equipment.

Personnel: includes military and civilian expenditure and pensions.

Infrastructure: includes NATO common infrastructure expenditure and national military construction

Others: includes operations and maintenance expenditure, others R&D expenditure

## 4. Empirical study

For each of these five groups, stationarity, the model estimation, and its validity, will be analysed through the Sargan test or the non-autocorrelation of the residuals depending on the robustness of the estimator. The descriptive statistics summary can be seen in Table 3.

**Table 3:** Summary statistics.

***LnGDP***	**GROUP**	**Mean**	**Std. Dev.**	**Min**	**Max**	**N**	**n**	**T**
** **	NATO countries	26,22	1,92	21,54	30,65	392	28	14
	Defeated & Neutral countries WWII	27,83	1,04	26,01	29,00	56	4	14
	Nuclear countries	29,22	0,86	28,42	30,65	43	3	14
	Easter European countries formerly linked to Soviet Union	24,71	1,23	21,54	27,10	182	13	14
	Conflict countries	26,81	0,59	26,00	27,58	28	2	14
	Rest countries	26,74	1,05	24,34	28,24	84	6	14
***LnMilExp***	**GROUP**	**Mean**	**Std. Dev.**	**Min**	**Max**	**N**	**n**	**T**
	NATO countries	22,02	2,07	17,86	27,29	392	28	14
	Defeated & Neutral countries WWII	23,60	0,90	21,99	24,62	56	4	14
	Nuclear countries	25,58	1,13	24,56	27,29	42	3	14
	Easter European countries formerly linked to Soviet Union	20,46	1,22	17,86	23,17	182	13	14
	Conflict countries	23,08	0,51	22,30	23,67	28	2	14
	Rest countries	22,19	1,35	19,22	23,77	84	6	14

Finally, a statistic test is proposed to determine if there is just one model or if it is possible identify different models across the countries, and in the latest to recognize in which one is the effect higher.

### 4.1 Dynamic panel data model NATO countries

Regarding the stationarity, the hypotheses tested were H_0_(1) and H_0_(2), where LnGDP and LnMilExp, respectively, are non-stationary panel data, containing unit root problems. As shown in Table 4, the logarithm of GDP and MilExp were stationary (H_0_(1) and H_0_(2) are rejected).

**Table 4:** Levin–Lin–Chu unit root test.

H_0_: Panels contain unit roots				
H_a_: Panels are stationary				
	**LnGDP**		**LnMilExp**	
	**Statistic**	**pvalue**	**Statistic**	**pvalue**
Unadjusted t	-10,233		-12,2105	
Adjusted t*	-5,7179	0,000	-5,496	0,000

The results obtained using this technique concludes that LnGDP is affected by the LnMilExp in lag one and four (see Table 5).

**Table 5:** Dynamic panel-data estimation.

Number of obs	280	
Number of groups	28	
Wald chi2 (6)	574,22	
pvalue	0	
	**Coef**	**pvalue**
LnGDP		
L1	0,382	0,000
L2	-0,127	0,000
L3	0,104	0,007
LnMilExp		
..	0,488	
L1	-0,313	0,000
L4	0,068	0,000
cons	11,689	0,000

Once the model has been estimated, it is necessary to ensure the autocorrelation conditions. The Arellano- Bond estimator only allows correlation in the residues in order 1. As shown in Table 6, the null hypothesis is accepted. The model is valid.

**Table 6:** Arellano -Bond test for zero autocorrelation in first differenced errors.

Order	z	Pvalue
1	-2,412	0,015
2	-1,114	0,265
3	1,441	0,149
4	-1,342	0,179
5	-0,553	0,58
6	-1,337	0,181
7	1,739	0,081
8	0,476	0,633
9	.	
10	.	

### 4.2 Dynamic panel data model defeated & neutral countries WWII

With respect to Germany, Italy, Spain and Portugal, the main highlights are the stationarity of their variables (see Table 7), and the validity of the model which concludes the positive impact of the Military expenditure in the GDP (see Tables 8 and 9).

**Table 7:** Levin–Lin–Chu unit root test.

H_0_: Panels contain unit roots				
H_a_: Panels are stationary				
	**LnGDP**		**LnMilExp**	
	**Statistic**	**pvalue**	**Statistic**	**pvalue**
Unadjusted t	-6,326		-4,694	
Adjusted t*	-3,133	0,001	-2,213	0,013

**Table 8:** Dynamic panel-data estimation.

Number of obs	44	
Number of groups	4	
Wald chi2 (3)	162,3	
pvalue	0	
	**Coef**	**pvalue**
LnGDP		
L1	0,024	0,775
L2	-0,164	0,011
LnMilExp	0,526	0,000
cons	19,3116	0,000

**Table 9:** Sargan test.

H_0_: Over identifying restrictions are valid	
chi (19)	44,548
Pvalue	0,183

### 4.3 Dynamic panel data model nuclear countries

Regarding the countries that are considered as nuclear countries, the variable LnGDP must be transformed due to the non-stationarity. Taking the first difference the variable becomes stationarity (see Table 10). Military expenditure even though affects negatively in the first year, is positive during the third year (see Table 11). According with the autocorrelation test, the model is well specified (see Table 12).

**Table 10:** Levin–Lin–Chu unit root test.

H_0_: Panels contain unit roots						
H_a_: Panels are stationary						
	**LnGDP**		**DLnGDP**		**LnMilExp**	
	Statistic	**pvalue**	**Statistic**	**pvalue**	**Statistic**	**pvalue**
Unadjusted t	-0,248		-7,656		-5,010	
Adjusted t*	0,299	0,618	-5,613	0,000	-3,163	0,001

**Table 11:** Dynamic panel-data estimation.

Number of obs	27	
Number of groups	3	
Wald chi2 (2)	196,14	
pvalue	0,000	
	**Coef**	**pvalue**
DLnGDP		
L1	0,545	0,76
L2	0,108	0,2
L3	-0,105	0,006
LnMilExp		
..	0,978	0,000
L1	-1,044	0,000
L3	0,465	0,000
cons	-10,201	0,000

**Table 12:** Arellano—Bond test for zero autocorrelation in first differenced errors.

H_0_: no autocorrelation		
**Order**	**z**	**pvalue**
1	-1,726	0,084
2	-0,959	0,337
3	1,555	0,198
4	-0,673	0,500
5	-0,015	0,987
6	-1,157	0,247
7	0,582	0,560
8	0,611	0,540
9	.	
10	.	

### 4.4 Dynamic panel data model Eastern European countries formerly linked to Soviet Union

Concerning the countries that were below the soviet orbit, the variables LnGDP and LnMilExp were stationarity (see Table 13). It is interesting to note that in this model the military expenditure has a positive impact in the years nine and ten (see Table 14). According with the Sargan test, the model is well estimated (see Table 15).

**Table 13:** Levin–Lin–Chu unit root test.

H_0_: Panels contain unit roots				
H_a_: Panels are stationary				
	**LnGDP**		**LnMilExp**	
	**Statistic**	**pvalue**	**Statistic**	**pvalue**
Unadjusted t	-13,757		-7,674	
Adjusted t*	-8,523	0,000	-2,455	0,007

**Table 14:** Dynamic panel-data estimation.

Number of obs	26	
Number of groups	13	
wald chi2 [6]	1494	
pvalue	0,000	
	**Coef**	**pvalue**
LnGDP		
L1	0,881	0,000
L4	0,290	0,019
L5	-0,334	0,001
LnMilExp		
L9	-0,6284	0,068
L10	0,141	0,000
L11	0,046	0,204
cons	1,557	0,675

**Table 15:** Sargan test.

H_0_: Over identifying restrictions are valid	
chi [19]	18,988
Pvalue	0,457

### 4.5 Dynamic panel data model conflict countries

With reference to the countries who are in conflict, the variables LnGDP and LnMilExp were stationarity (see Table 16) and there is a negative impact during the first year in the economy (see Table 17). According with the Sargan test, the model is well estimated (see Table 18).

**Table 16:** Levin–Lin–Chu unit root test.

H_0_: Panels contain unit roots				
H_a_: Panels are stationary				
	**LnGDP**		**LnMilExp**	
	**Statistic**	**pvalue**	**Statistic**	**pvalue**
Unadjusted t	-3,218		-3,283	
Adjusted t*	-1,662	0,048	-1,956	0,025

**Table 17:** Dynamic panel-data estimation.

Number of obs	24	
Number of groups	2	
Wald chi2 [3]	179,82	
pvalue	0,000	
	**Coef**	**pvalue**
LnGDP		
L1	0,580	0,000
LnMilExp		
..	0,636	0,000
L1	-0,310	0,099
cons	3,727	0,046

**Table 18:** Sargan test.

H_0_: Over identifying restrictions are valid	
chi [19]	23,514
pvalue	0,317

### 4.6 Dynamic panel data model rest countries

As for the rest of allies’ countries, the variables LnGDP and LnMilExp were stationarity (see Table 19) and there is a negative impact during the second year in the economy (see Table 20). According with the Sargan test, the model is well estimated (see Table 21).

**Table 19:** Levin–Lin–Chu unit root test.

H_0_: Panels contain unit roots				
H_a_: Panels are stationary				
	**LnGDP**		**LnMilExp**	
	**Statistic**	**pvalue**	**Statistic**	**pvalue**
Unadjusted t	-8,007		-6,198	
Adjusted t*	-4,782	0,000	-2,506	0,006

**Table 20:** Dynamic panel-data estimation.

Number of obs	48	
Number of groups	6	
Wald chi2 [5]	120,84	
pvalue	0,000	
	**Coef**	**pvalue**
LnGDP		
L1	0,532	0,000
L4	-0,476	0,000
L5	0,217	0,004
LnMilExp		
..	0,460	0,000
L2	-0,322	0,000
cons	16,431	0,000

**Table 21:** Sargan test.

H_0_: Over identifying restrictions are valid	
chi [19]	39,215
Pvalue	0,550

### 4.7 Model selected: Poolability test

Once the six models have been examined, the next question is to determine if only one data panel model exists for the twenty eight countries or if it is possible to have different behaviours within the group, particularly if it is possible to group in five.

For this purpose, the statistic proposed is based on the Hsiao homogeneity test for linear regression models [82] whose statistics is:

$$F=\frac{\frac{\left(S_{3}-S_{1}\right)}{\left(N-1)(K-1\right)}}{\frac{S_{1}}{NT-N(K+1)}}$$ (1)

where S_3_ are the sums of squared residuals unique panel data model, and S1 are the sums of squared residuals of panel data models. Therefore, rejecting the null hypothesis is to accept that the no homogeneity is in the intercept.

$$H_{0}:\;\alpha_{1}=\alpha_{2}=\cdots \;\alpha_{N}$$

$$\beta_{1}=\beta_{2}=\cdots \;\beta_{N}$$

In order to determine if it is more convenient to create five panel data model according with the historical characteristics of the countries rather than having only one group, this papers compares the sum of the squared residuals of a unique panel data against the alternative hypothesis of not being possible to pool all the data together. The difference with the Hsiao test is that it starts from estimating the model with the same functional form and with the same explanatory variable.

Based on Hsiao test, the statistic proposed, once the residuals have been typified, is:

$$F=\frac{\frac{\left(S_{3}-S_{1}\right)}{\left(N_{11}K_{11}+N_{12}K_{12}+N_{13}K_{13}{+N}_{14}K_{14}+N_{15}K_{15})-(K_{11}+K_{12}+K_{13}+K_{14+}K_{15})-{(N}_{11}+N_{12}+N_{13}{+N}_{14}+N_{15})+1\right)}}{\frac{S_{1}}{NT-(N_{11}K_{11}+N_{12}K_{12}+N_{13}K_{13}{+N}_{14}K_{14}+N_{15}K_{15}+N)}}$$ (2)

where S3 are the sums of squared residuals typified unique panel data model, and S1 are the sums of squared residuals typified 5 panel data models.

The result is an F-distribution with 117 parameters and 80 degrees of freedom (5%) being the value 0,2881.

This value is compared with 1,41265 resulting from a F117,80 (5%), rejecting the null hypothesis and being better to not to be pooled all the data together. This result is consistent with the differences that exist across the countries in economic terms. Therefore, it could be possible to question if this positive impact is higher in the nuclear countries than in the others group.

To determine if this effect is higher in the nuclear countries, the regression coefficients between models will be compared [83, 84].

Therefore, the hypotheses tested were:

H_0_: = β _(nuclear countries)_ = β _(Defeated & Neutral countries WWII)_,

H_1_: = β _(Defeated & Neutral countries WWII)_ > β _(Defeated & Neutral countries WWII)_

And the statistical contrast performs is:

$$Z=\frac{b_{1}-b_{2}}{\sqrt{SEb_{1}^{2}+SEb_{2}^{2}}}$$ (3)

where:

b_1_ is regression coefficient 1,

b_2_ is regression coefficient 2,

SEb_1_^2^ is standard deviation of regression coefficient 1, and

SEb_2_^2^ is standard deviation of regression coefficient 2.

According with formula 3, the result of the statistical contrast is 5,606 and the p value associates is 5,97529E-08, which means that H_0_ is rejected and that the coefficient which measures the influence of the LnMilExp in the explain variable is higher in the nuclear countries than in the defeated and neutral countries WWII.

If this contrast is reapplied to the rest of the groups, it can be concluded that the positive influence of the military expenditure is higher in the nuclear than in the rest of the groups (see Table 22), while it is not possible to affirm the contrary statement.

**Table 22:** Statistical contrast.

*Statistic/ (pvalue)*			
	*Defeated & Neutral countries WWII*	*Conflict countries*	*Rest countries*
Nuclear countries	5,606	2,419	6,052
	(-0,000)***	(-0,021)***	(-0,000)***
Defeated & Neutral countries WWII		0,815	8,72E-01
		(-0,286)	(-0,272)
Conflict countries			1,276
			(-0,176)

***significant at the 0,5% level

## 5. Results and discussion

Once the empirical study has been carried out, it is possible to summarize that all the variables are stationary so the relationship between them is stable in the long run. Table 23 shows the main results of the panel analysis conducted. Overall, it is possible to conclude that during the first year, there is a positive impact of the military expenditure in the GDP of the countries analysed, which becomes blurred within the following years.

**Table 23:** Comparative of results dynamic panel data model.

	*NATO Countries*	*Defeated& Neutral countries WWII*	Nuclear countries*	*Easter European countries formerly linked to Soviet Union*	*Conflict countries*	*Rest countries*
***LnGDP***	**Coef. Robust**	**Coef.**	**Coef. Robust**	**Coef.**	**Coef.**	**Coef.**
L1.	0,382	0,024	0,054	0,881	0,580	0,532
L2.	-0,127	-0,164	0,108			
L3.	0,104		-0,105			
L4.				0,290		-0,476
L5.				-0,334		0,217
**LnMilExp**	**Coef. Robust**	**Coef.**	**Coef. Robust**	**Coef.**	**Coef.**	**Coef.**
-	0,480	0,526	0,978		0,636	0,460
L1.	-0,313		-1,044		-0,310	
L2.						-0,322
L3.			0,465			
L4.	0,068					
L9.				-0,062		
L10.				0,141		
L11.				0,046		
_cons	11,689	19,311	-10,201	1,557	3,727	16,431

*DLnGDP

These results provide a new perspective for all the countries that are part of the Alliance in such a way they are able to value the impact on their economies in case of increasing the military expenses up to the 2% that was established during the Wales Summit in 2014.

Moreover, thanks to these outcomes, the investment in defence could be perceived by the society as an important public good that impacts positively in the economy. The Western societies of the NATO members might not assume that the widespread security feeling may be maintained without making a higher commitment to invest in defence, even less if USA would like to reduce its role in NATO. There is no doubt that the instability in some regions of the world, together with the progress in technology have increased the catalogue of risks and threats, which could become material if a suitable investment in defence is not taken into account. At the same time, countries ranked by their high potential military strength, such as China and Russia, increase their defence budgets year by year, in order to sustain their international strategies in sensitive areas such the Mediterranean Sea, the China Sea, the Middle East or countries in the Eastern European region as Belarus or Ukraine among others.

Therefore, the results, which highlight the positive impact of defence expenditure in the economies of the NATO countries, confirm empirically the relevance and the need of investing in defence in order to strengthen the feeling of security and contribute to welfare society.

## 6. Implications and concluding remarks

Numerous authors have studied the effect of defence expenditure on the economy growth. From Benoit in 1973 to date, these variables have been analysed to verify their relationship without arriving to a single answer. The results are undetermined and contradictory, being able to distinguish four possible effects: (a) positive, (b) negative, (c) absence of relationship, and (d) effects that the growth of the economy produces on defence expenditure.

For the NATO case, even though it is possible to find some papers such as Ozun [5], whose findings show the existence of a relationship between economic growth and defence spending in seven NATO countries, Odehnal et al. [3, 4] who study the economic environment as a determinant of military expenditure, or Spangler [6] focused on the European military expenditure in relation with the US expenditure. None of them investigate both the relationship between military expenditure and the economic growth with a dynamic panel data model for the NATO countries and the confirmation of five groups of countries inside the Alliance.

The present study expands the results exposed by Ozun [5]. Ozun’s paper [5] covers the period between 1949 and 2006, so that the influence of the Easter countries is minimum due to most of them belonged to NATO in 2004.This study is focused on determining if there is a relationship between military expenditure and economic growth in the period 2005-2018and uses the Arellano-Bond dynamic panel data model. In addition, it analyses if it is possible to find a unique model, or if it is possible to identify several, through the poolability test.

Even though this model concluded with the positive impact of the military expenditure to the GDP, this paper would be a relevant contribution to determine if the twenty eight NATO countries behaviours are unique or if it is possible to identify different groups according with their characteristics. The results confirm that it is feasible to identify the existences of five different groups across the NATO countries. In addition, it could be affirmed that the impact is higher in the nuclear countries than in the rest of the groups.

As has been shown in this study of dynamic panel modelling, the positive effect of defence spending on a country's economy corroborates, in an empirical way, the relevance of our societies continuing to invest in defence, both to strengthen the sense of security and because it contributes to growth and benefits the society due to the great influence of the country's economic environment on its military spending.

In view of these, it seems logical to think about the requirement of a stable defence policy that ensures its financing and that allows the achievement of the necessary capacities to comply with national security requirements and international commitments.

Finally, given the complex reality of defence spending in NATO countries, it could be interesting, as a potential avenue for future research, to investigate the transmission channels of the positive effects of defence spending on economic growth, in the countries where there are, of course.

## Supporting information

S1 Fig Military expenditure as a share of GDP. (PDF)

S2 Fig Military expenditure as a share of GDP from 2005 to 2018. (PDF)

S1 Data Data base file downloaded from the World Bank open data https://data.worldbank.org/. (XLSX)

## References

[1] The Washington Times. Donald Trump questions NATO's usefulness in post-Cold War era. [Internet]. Washington D.C.—[cited 2016 Mar 28]. Available from: https://www.washingtontimes.com/news/2016/mar/28/donald-trump-nato-very-obsolete/

[2] North Atlantic Treaty Organization. Wales Summit Declaration. Issued by the Heads of State and Government participating in the meeting of the North Atlantic Council in Wales. [Internet]. (Retrieved May 25, 2020 from https://www.nato.int/cps/en/natohq/official_texts_112964.htm

[3] Odehnal J, Neubauer J. Economic, Security, and Political Determinants of Military Spending in NATO Countries. Def. Peace Econ. 2018; 1–15. 10.1080/10242694.2018.1544440

[4] Odehnal J. Military expenditures and free-riding in NATO. Peace Econ. Peace Sci. Public Policy. 2015; 21(4): 479–487. 10.1515/peps-2015-0015

[5] Ozun A. Erbaykal E. Further evidence on defence spending and economic growth in NATO countries. Koc University-TUSIAD Economic Research Forum Working Paper Series. 2011; No. 1119.

[6] Spangler E. Allies with benefits: US effect on European demand for military expenditures. Def. Peace Econ. 2018; 29(7): 731–747. 10.1080/10242694.2017.1310365

[7] Benoit E. Defense and economic growth in developing countries. Lexington: Lexington Books; 1973.

[8] Benoit E. Growth and defense in developing countries. Econ. Dev. Cult. Change. 1978; 26(2): 271–280. 10.1086/451015

[9] Deger S, Sen S. Military expenditure and developing countries. In: Hartley K, Sandler T, editors. Handbook of Defense Economics Elsevier; 1995; 1. pp. 275–307. 10.1016/S1574-0013(05)80013-4

[10] Dunne JP, Tian N. Military expenditure and economic growth: A survey. The Economics of Peace and Security Journal, 2013; 8(1): 5–11. 10.15355/epsj.8.1.5

[11] Dunne JP, Uye M. Military Spending and Development. In: Andrew T, editor. The Global Arms Trade. London, UK: Europe/Routledge; 2010 p. 293–305.

[12] Emmanouilidis K, Karpetis C. The Defense–Growth Nexus: A Review of Time Series Methods and Empirical Results. Def. Peace Econ. 2018; 1–18. 10.1080/10242694.2018.1428261

[13] Ram R. Defense expenditure and economic growth. In: Hartley K, Sandler T, editors. Handbook of Defense Economics Elsevier; 1995; 1. pp. 251–274. 10.1016/S1574-0013(05)80012-2

[14] Smith RP. Military expenditure and investment in OECD countries, 1954–1973. J. Comp. Econ. 1980; 4(1): 19–32. 10.1016/0147-5967(80)90050-5

[15] Faini R, Annez P, Taylor L. Defense spending, economic structure and growth: Evidence among countries and over time. Econ. Dev. Cult. Change. 1984; 32(3): 487–498. 10.1086/451402

[16] Chan S. Military expenditures and economic performance. In: United States Arms Control and Disarmament Agency, editor. World military expenditures and arms transfers. Washington, DC: United States Arms Control and Disarmament Agency; 1986 pp. 29–38.

[17] Yakovlev P. Arms trade, military spending, and economic growth. Def. Peace Econ. 2007; 18(4): 317–338. 10.1080/10242690601099679

[18] Deger S, Sen S. Military expenditure, spin-off and economic development. J. Dev. Econ. 1983; 13(1–2): 67–83. 10.1016/0304-3878(83)90050-0

[19] Antonakis N. Military expenditure and economic growth in Greece, 1960–90. J. Peace Res. 1997; 34(1): 89–100. 10.1177/0022343397034001007

[20] Galvin H. The impact of defence spending on the economic growth of developing countries: A cross-section study. Def. Peace Econ. 2003; 14(1): 51–59. 10.1080/10242690302932

[21] Feder G. On exports and economic growth. J. Dev. Econ. 1983; 12(1–2): 59–73. 10.1016/0304-3878(83)90031-7

[22] Atesoglu HS, Mueller MJ. Defence spending and economic growth. Def. Peace Econ.1990; 2(1): 19–27. 10.1080/10430719008404675

[23] Ram R. Government Size and Economic Growth: A new framework and some evidence from cross-section and time-series data. Am. Econ. Rev. 1986; 76(1): 191–203.

[24] Ward MD, Davis D, Penubarti M, Rajmaira S, Cochran M. Country Survey I–Military Spending in India. Def. Peace Econ. 1991; 3(1): 41–63

[25] Biswas B, Ram R. Military expenditures and economic growth in less developed countries: An augmented model and further evidence. Econ. Dev. Cult. Change. 1986; 34(2): 361–372. 10.1086/451533

[26] Alexander WRJ. The Impact of Defence Spending on Economic Growth: A multi‐sectorial approach to defence spending and economic growth with evidence from developed economies. Def. Peace Econ. 1990; 2(1): 39–55. 10.1080/10430719008404677

[27] Huang C, Mintz A. Ridge regression analysis of the defence‐growth trade-off in the United States. Def. Peace Econ. 1990; 2(1): 29–37. 10.1080/10430719008404676

[28] Huang C, Mintz A. Defence expenditures and economic growth: The externality effect. Def. Peace Econ. 1991; 3(1): 35–40. 10.1080/10430719108404713

[29] Adams FG, Behrman JR, Boldin M. Government expenditures, defense, and economic growth in the LDCs: A revised perspective. Confl. Manage. Peace Sci.1991; 11(2): 19–35. 10.1177/073889429101100202

[30] Nikolaidou E. Military spending and economic growth in Greece, A multi-sector analysis, 1961–1996. Department of Economics; Middlesex University Business School; 1998.

[31] Aizenman J, Glick R. Military expenditure, threats, and growth. J. Int. Trade Econ. Dev.2006; 15(2): 129–155. 10.1080/09638190600689095

[32] Dunne JP, Nikolaidou E. Defence spending and economic growth in the EU15. Def. Peace Econ. 2012; 23(6): 537–548. 10.1080/10242694.2012.663575

[33] Hou N, Chen B. Military expenditure and economic growth in developing countries: Evidence from system GMM estimates. Def. Peace Econ. 2013a; 24(3): 183–193. 10.1080/10242694.2012.710813

[34] Hou N, Chen B. Military Expenditure and Economic Growth in South Asia. In Li JS, Chen B, Hou N, editors. Cooperation for a Peaceful and Sustainable World Part 2 (Contributions to Conflict Management, Peace Economics and Development, Volume 20). Emerald Group Publishing Limited; 2013b. p. 213–223.

[35] Hou N, Chen B. Military expenditure and investment in OECD countries: Revisited. Peace Econ. Peace Sci. Public Policy, 2014; 20(4): 621–630. 10.1515/peps-2014-0031

[36] Deger S, Smith R. Military expenditure and growth in less developed countries. J. Confl. Resolut. 1983; 27(2): 335–353. 10.1177/0022002783027002006

[37] Deger S. Economic development and defense expenditure. Econ. Dev. Cult. Change. 1986a; 35(1): 179–196.

[38] Deger S. Military expenditure in third world countries: The economic effects. Taylor & Francis. 1986b.

[39] Scheetz T. The macroeconomic impact of defence expenditures: Some econometric evidence for Argentina, Chile, Paraguay and Peru. Def. Peace Econ. 1991; 3(1): 65–81. 10.1080/10430719108404715

[40] Sezgin S. An empirical analysis of Turkey's defence‐growth relationships with a multi‐equation model (1956–1994). Def. Peace Econ. 2001; 12(1): 69–86. 10.1080/10430710108404977

[41] Chowdhury AR. A causal analysis of defense spending and economic growth. J. Confl. Resolut. 1991; 35(1): 80–97. 10.1177/0022002791035001005

[42] Seiglie C, Liu PC. Arms races in the developing world: Some policy implications. J. Policy Model. 2002; 24(7–8): 693–705. 10.1016/S0161-8938(02)00165-5

[43] Pradhan RP. Modelling the nexus between defense spending and economic growth in asean-5: Evidence from co-integrated panel analysis. Afr. J. Pol. Sci. Int. Relat. 2010; 4(8): 297–307.

[44] Balan F. The Nexus between Political Instability Defence Spending and Economic Growth in the Middle East Countries: Bootstrap Panel Granger Causality Analysis. Petroleum-Gas University of Ploiesti Bulletin, Technical Series. 2015; 67(4): 1–14.

[45] Su C, Xu Y, Ling H, Lobont OR, Liu Z. Dynamic causalities between defense expenditure and economic growth in China: evidence from rolling granger causality test. Def. Peace Econ. 2018; 1–18. 10.1080/10242694.2018.1505583

[46] Yildirim J, Sezgin S, Öcal N. Military Expenditure and Economic Growth in Middle Eastern Countries: A dynamic panel data analysis. Def. Peace Econ. 2005; 16(4): 283–295. 10.1080/10242690500114751

[47] Dunne JP, Nikolaidou E, Chiminya A. Military Spending, Conflict and External Debt in Sub-Saharan Africa. Def. Peace Econ. 2019;30(4): 462–473. 10.1080/10242694.2018.1556996

[48] Cappelen Â, Gleditsch NP, Bjerkholt O. Military spending and economic growth in the OECD countries. J. Peace Res. 1984; 21(4): 361–373. 10.1177/002234338402100404

[49] Landau D. Is one of the ‘peace dividends’ negative? Military expenditure and economic growth in the wealthy OECD countries. Quarterly Review of Economics and Finance. 1996; 36(2): 183–195. 10.1016/S1062-9769(96)90038-1

[50] Lee C, Chen S. Do defence expenditures spur GDP? A panel analysis from OECD and non‐OECD countries. Def. Peace Econ. 2007; 18(3): 265–280. 10.1080/10242690500452706

[51] Joerding W. Economic growth and defense spending: Granger causality. J. Dev. Econ. 1986; 21(1): 35–40. 10.1016/0304-3878(86)90037-4

[52] Dakurah AH, Davies SP, Sampath RK. Defense spending and economic growth in developing countries: A causality analysis. J. Policy Model. 2001; 23(6): 651–658. 10.1016/S0161-8938(01)00079-5

[53] D’Agostino G, Dunne JP, Pieroni L. Military expenditure, endogeneity and economic growth. Def. Peace Econ. 2019; 30(5): 509–524. 10.1080/10242694.2017.142231

[54] Lebovic JH, Ishaq A. Military Burden, Security Needs, and Economic Growth in the Middle East. J. Confl. Resolut. 1987; 31(1): 106–138. 10.1177/0022002787031001007

[55] Pan C, Chang T, Wolde-Rufael Y. Military spending and economic growth in the Middle East countries: Bootstrap panel causality test. Def. Peace Econ. 2015; 26(4): 443–456. 10.1080/10242694.2014.891356

[56] Coutts A, Daoud A, Fakih A, Marrouch W, Reinsberg B. Guns and butter? Military expenditure and health spending on the eve of the Arab Spring. Def. Peace Econ. 2019; 30(2): 227–237. 10.1080/10242694.2018.1497372

[57] Kollias C, Manolas G, Paleologou S. Defence Expenditure and Economic Growth in the European Union: A causality analysis. J. Policy Model. 2004; 26(5): 553–569. 10.1016/j.jpolmod.2004.03.013

[58] Kollias C, Mylonidis N, Paleologou S. A Panel Data Analysis of the nexus between Defence Spending and Growth in the European Union. Def. Peace Econ. 2007; 18(1): 75–85. 10.1080/10242690600722636

[59] Mylonidis N. Revisiting the nexus between military spending and growth in the European Union. Def. Peace Econ. 2008; 19(4): 265–272. 10.1080/10242690802164801

[60] Chang T, Lee C, Chu H. Revisiting the Defense–Growth nexus in European Countries. Def. Peace Econ. 2015; 26(3): 341–356. 10.1080/10242694.2013.832556

[61] Hunter E. The demand for military expenditure in Europe: the role of fiscal space in the context of a resurgent Russia. Def. Peace Econ. 2017; 30(1): 72–84. 10.1080/10242694.2017.1373542

[62] Michael C, Stelios R. Defense spending and unemployment. Evidence from southern European countries. Peace Econ. Peace Sci. Public Policy. 2017; 23(1): 1–36. 10.1515/peps-2016-0026

[63] Daddi P, D’Agostino G, Pieroni L. Does military spending stimulate growth? An empirical investigation in Italy. Def. Peace Econ. 2018; 29(4): 440–458. 10.1080/10242694.2016.1158438

[64] Dimitraki O, Kartsaklas A. Sovereign debt, deficits and defence spending: the case of Greece. Def. Peace Econ. 2018; 29(6): 712–727. 10.1080/10242694.2017.1289497

[65] Aben J, Fontanel J. Military Expenditure as a Proxy for State Power. The Case of France. Def. Peace Econ.2019; 30(2): 133–141. 10.1080/10242694.2018.1460714

[66] Berg H, Ofstad A, Øhrn M. Military off the shelf procurements: A Norwegian case study. Def. Peace Econ. 2019; 30(1): 98–110. 10.1080/10242694.2017.1342182

[67] Blomberg SB. Growth, political instability, and the defense burden. Board of Governors of the Federal Reserve System International Finance Discussion Papers. 1992; No. 436.

[68] D’Agostino G, Dunne JP, Pieroni L. Corruption, military spending and growth. Def. Peace Econ. 2012; 23(6): 591–604. 10.1080/10242694.2012.663579

[69] Ali H, Adebola S. Military spending, corruption, and the welfare consequences. Def. Peace Econ. 2019; 1–15. 10.1080/10242694.2019.1567181

[70] Solarin SA. Determinants of military expenditure and the role of globalisation in a cross-country analysis. Def. Peace Econ.2018; 29(7): 853–870. 10.1080/10242694.2017.1309259

[71] Xu Y, Wei C, Tao R. Is defense spending inflationary? Time–frequency evidence from China. Def. Peace Econ. 2018; 1–15. 10.1080/10242694.2018.1457197

[72] Fan H, Liu W, Coyte PC. Do military expenditures crowd-out health expenditures? Evidence from around the World, 2000–2013. Def. Peace Econ. 2018; 29(7): 766–779. 10.1080/10242694.2017.1303303

[73] Kishore R, Kabir E, Bin R. Investment in Research and Development compared to military expenditure: is research worthwhile? Def. Peace Econ. 2018; 1–12. 10.1080/10242694.2018.1477235

[74] Aziz N, Khalid U. Armed conflict, military expenses and FDI inflow to developing countries. Def. Peace Econ. 2019; 30(2): 238–251

[75] Bolzan L, Blackwell P. The Brazilian National Defence Strategy: Defence Expenditure Choices and Military Power. Def. Peace Econ. 2019; 1–16. 10.1080/10242694.2019.1588030

[76] Ahmed S, Alam K, Rashid A, Gow J. Militarisation, energy consumption, CO_2_ emissions and economic growth in Myanmar. Def. Peace Econ.2019; 1–27. 10.1080/10242694.2018.1560566

[77] Judson RA, Owen AL. Estimating dynamic panel data models: a guide for macroeconomists. Econ. Lett. 1999; 65(1): 9–15. 10.1016/S0165-1765(99)00130-5

[78] Nickell S. Correcting the Biases in Dynamic Models with Fixed Effects.Princeton University, Industrial Relations Section. Working paper. 1980; No. 133.

[79] Dunne JP, Perlo-Freeman S. The demand for military spending in developing countries: A dynamic panel analysis. Def. Peace Econ. 2003; 14(6): 461–474. 10.1080/1024269032000085224

[80] Arellano M, Bond S. Some tests of specification for panel data: Monte Carlo evidence and an application to employment equations. Rev. Econ. Stud.1991;58(2): 277–297. 10.2307/2297968

[81] Pesaran MH, Smith R. Estimating long-run relationships from dynamic heterogeneous panels.J Econom. 1995;68(1): 79–113. 10.1016/0304-4076(94)01644-F

[82] Hsiao C. Analysis of panel data. 54. Cambridge University Press; 2014.

[83] Clogg CC, Petkova E, Haritou A. Statistical methods for comparing regression coefficients between models. Am. J. Sociol. 1995; 100(5): 1261–1293. 10.1086/230638

[84] Paternoster R, Brame R, Mazerolle P, Piquero A. Using the correct statistical test for the equality of regression coefficients. Criminology. 1998; 36(4): 859–866.

