# Supplementary material for: A tale of five stories: Defence spending and economic growth in NATO´s countries
Source: PLoS One. 2021 Jan 11;16(1):e0245260. doi: 10.1371/journal.pone.0245260 (PMC7799761; doi:10.1371/journal.pone.0245260)
Supplement: S1 Fig — (PDF) [file pone.0245260.s001.pdf]

NATO OTAN | PUBLIC DIPLOMACY DIVISION / DIVISION DIPLOMATIE PUBLIQUE | *Press Release* / *Communiqué de presse*

25 June/Juin 2019 **COMMUNIQUE**
PR/CP(2019)069

Defence Expenditure of NATO Countries (2012-2019)

NATO collects defence expenditure data from Allies on a regular basis and presents aggregates and subsets of this information. Each Ally's Ministry of Defence reports current and estimated future defence expenditure according to an agreed definition of defence expenditure. The amounts represent payments by a national government actually made, or to be made, during the course of the fiscal year to meet the needs of its armed forces, those of Allies or of the Alliance. In the figures and tables that follow, NATO also uses up-to-date economic and demographic information available from the Directorate-General for Economic and Financial Affairs of the European Commission (DG-ECFIN), and the Organisation for Economic Co-operation and Development (OECD).

In view of differences between both these sources and national GDP forecasts, and also the definition of NATO defence expenditure and national definitions, the figures shown in this report may diverge considerably from those which are quoted by media, published by national authorities or given in national budgets. Equipment expenditure includes expenditure on major equipment as well as on research and development devoted to major equipment. Personnel expenditure includes pensions paid to retirees.

The cut-off date for information used in this report was 20 June 2019. Figures for 2018 and 2019 are estimates.

News and information is routinely placed on the NATO website. This includes audio files, transcripts and high resolution photographs, which are posted as soon as possible after events of media interest. Check the 'What's New' file.
Nouvelles et informations sont régulièrement affichées sur le site Web de l'OTAN, sous la forme de fichiers audio, de transcriptions et de photographies destinées à la publication. Elles sont disponibles le plus rapidement possible après les événements présentant un intérêt pour les médias. Cliquer sur "Quoi de neuf?"

North Atlantic Treaty Organisation – Organisation du Traité de l'Atlantique Nord
Press & Media – Presse & Médias
B-1110 Bruxelles Belgique
E-mail: press@hq.nato.int – Tel.: 32 2 707 50 41 – Fax: 32 2 707 13 99 – Internet: www.nato.int

Graph 1 : NATO Europe and Canada - defence expenditure

(annual real change, based on 2015 prices and exchange rates)

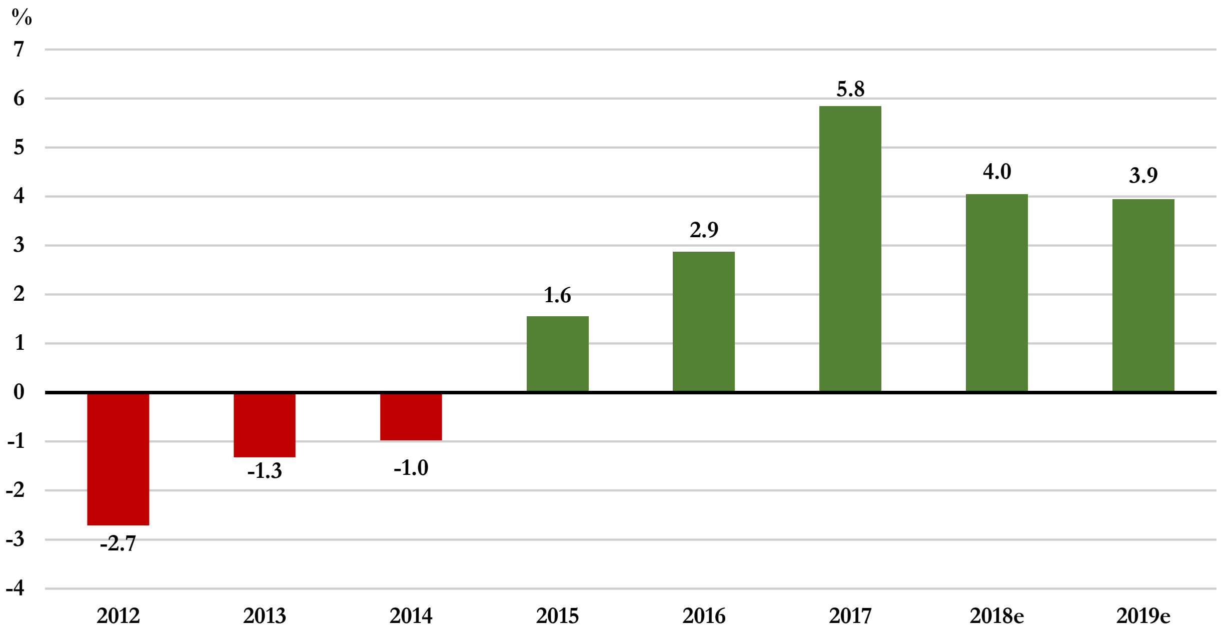

Notes: Figures for 2018 and 2019 are estimates. The NATO Europe and Canada aggregate from 2017 onwards includes Montenegro, which became an Ally on 5 June 2017.

Graph 2 : Defence expenditure as a share of GDP and equipment expenditure as a share of defence expenditure 2019e

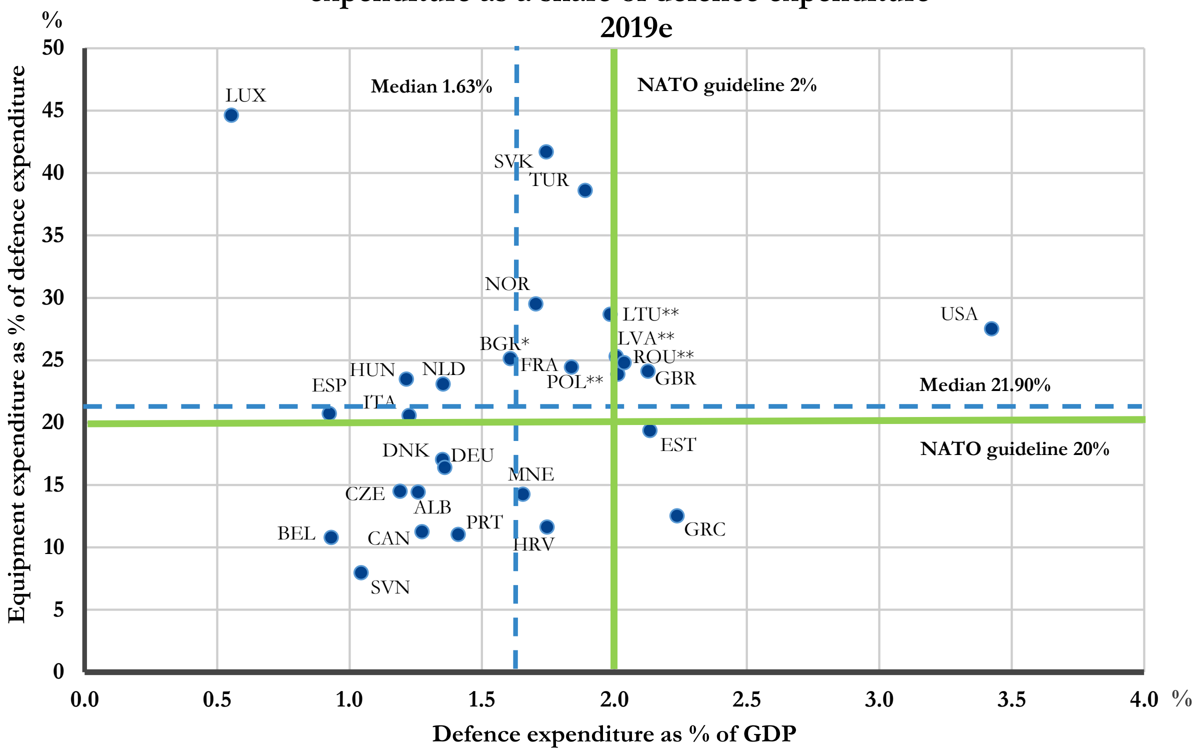

Notes: Figures for 2018 and 2019 are estimates. The NATO Europe and Canada aggregate from 2017 onwards includes Montenegro, which became an Ally on 5 June 2017.

* Defence expenditure does not include pensions.

** These Allies have national laws and political agreements which call for 2% of GDP to be spent on defence annually, consequently estimates are expected to change accordingly. For the past years, Allies' defence spending was based on the then available GDP data and Allies may, therefore, have met the 2% guideline when using those figures (In 2018, Lithuania met 2% using November 2018 OECD figures).

Graph 3 : Defence expenditure as a share of GDP (%)

(based on 2015 prices and exchange rates)

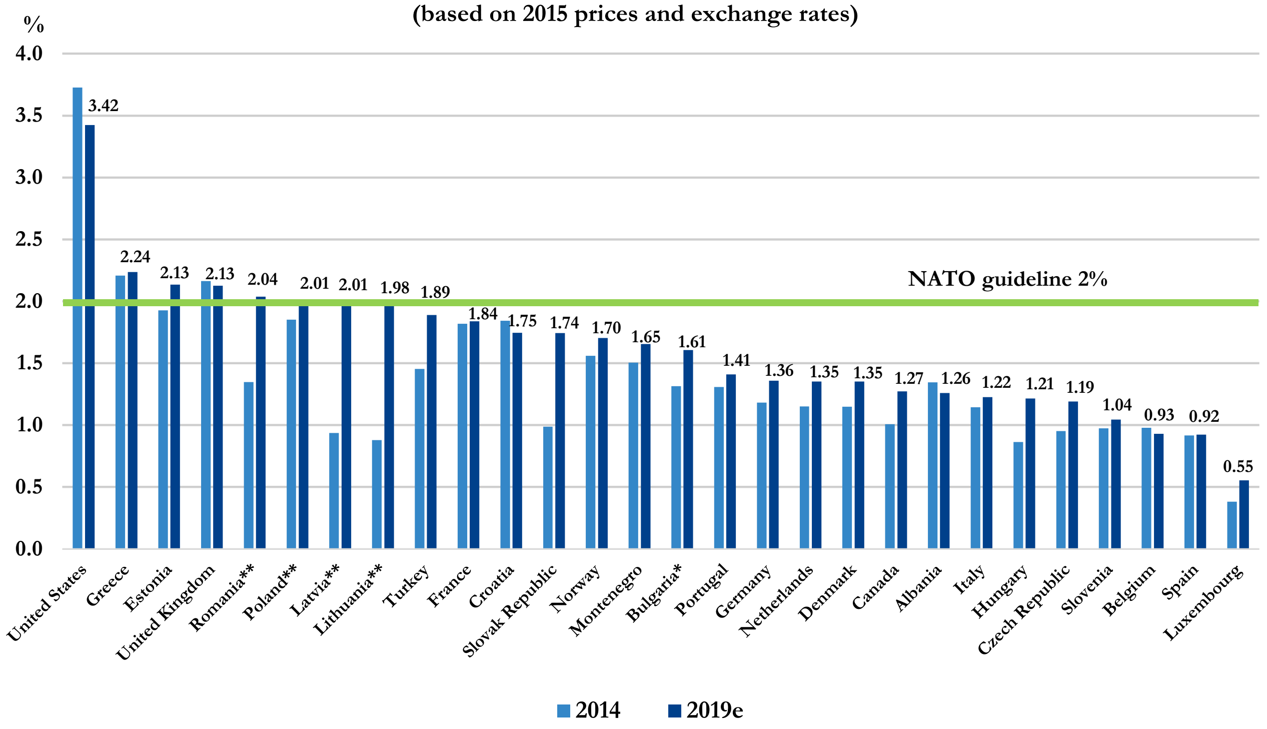

Notes: Figures for 2019 are estimates.

* Defence expenditure does not include pensions.

** These Allies have national laws and political agreements which call for 2% of GDP to be spent on defence annually, consequently estimates are expected to change accordingly. For the past years, Allies' defence spending was based on the then available GDP data and Allies may, therefore, have met the 2% guideline when using those figures (In 2018, Lithuania met 2% using November 2018 OECD figures).

Graph 4 : Equipment expenditure as a share of defence expenditure (%)

(based on 2015 prices and exchange rates)

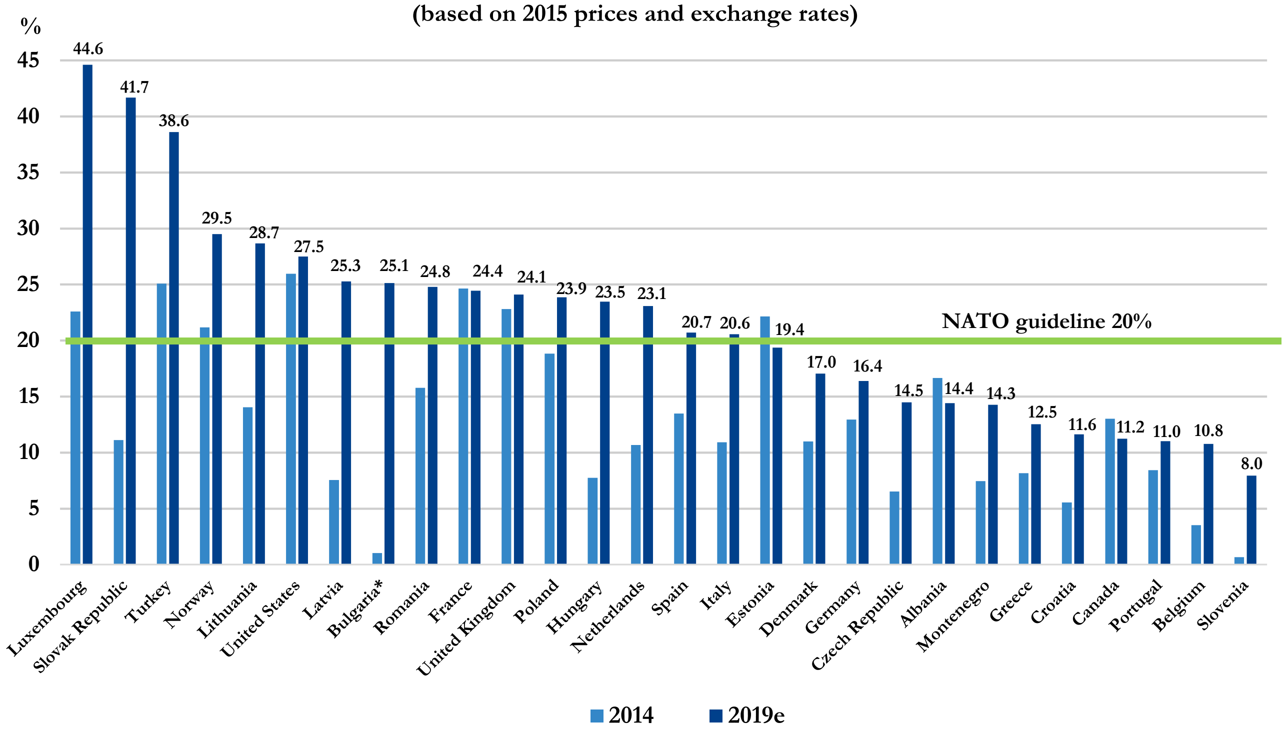

Notes: Figures for 2019 are estimates.

* Defence expenditure does not include pensions.

** These Allies have national laws and political agreements which call for 2% of GDP to be spent on defence annually, consequently estimates are expected to change accordingly. For the past years, Allies' defence spending was based on the then available GDP data and Allies may, therefore, have met the 2% guideline when using those figures (In 2018, Lithuania met 2% using November 2018 OECD figures).

Graph 5 : Defence expenditure as a share of GDP (%)
(based on 2015 prices and exchange rates)

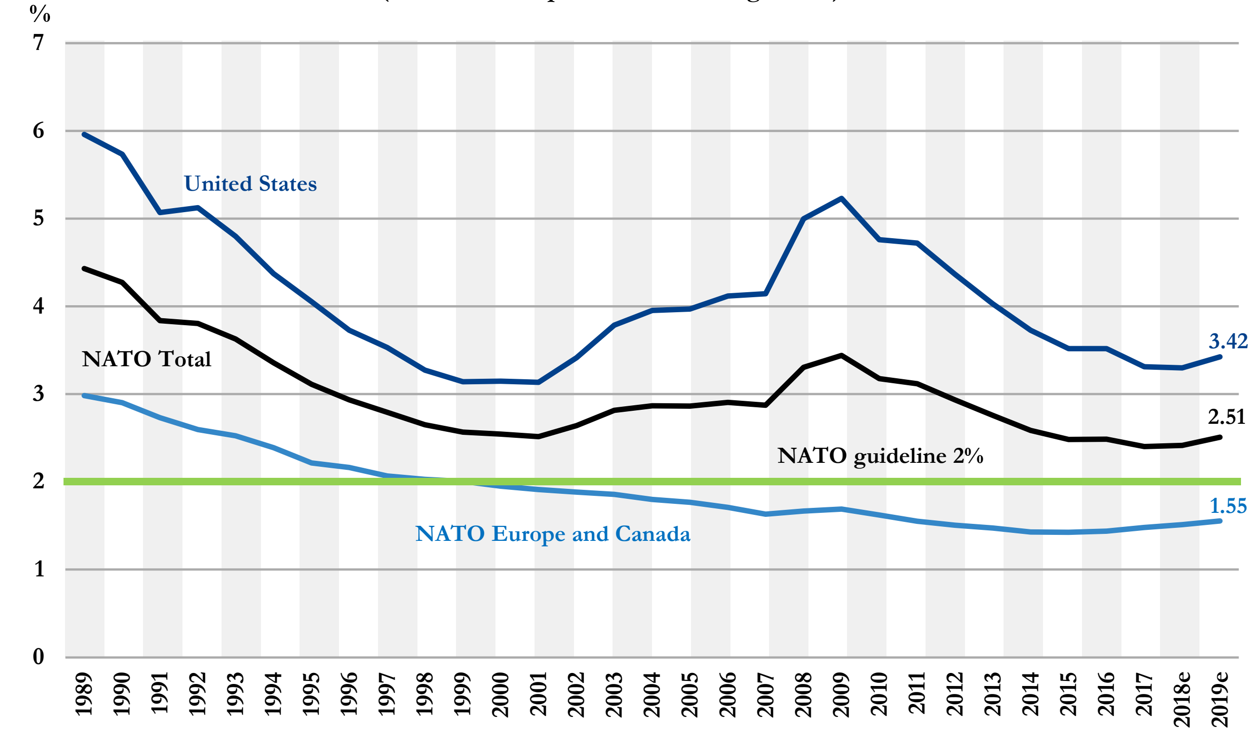

Notes: Includes enlargements which took place in: 1999 (3 Allies), 2004 (7 Allies), 2009 (2 Allies) and 2017 (1 Ally). Figures for 2018 and 2019 are estimates.

Graph 6 : NATO Europe and Canada - defence expenditure
(billion US dollars, based on 2015 prices and exchange rates)

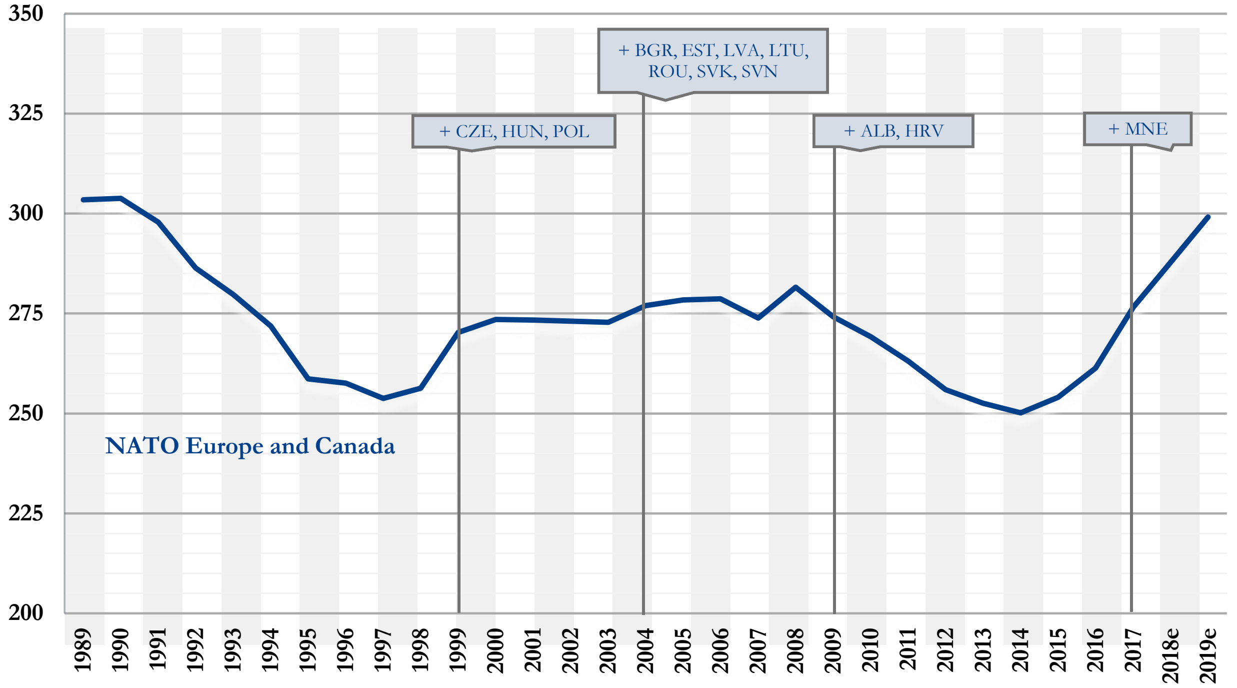

Notes: Includes enlargements which took place in: 1999 (3 Allies), 2004 (7 Allies), 2009 (2 Allies) and 2017 (1 Ally). Figures for 2018 and 2019 are estimates.

Graph 7 : Defence expenditure

(billion US dollars, based on 2015 prices and exchange rates)

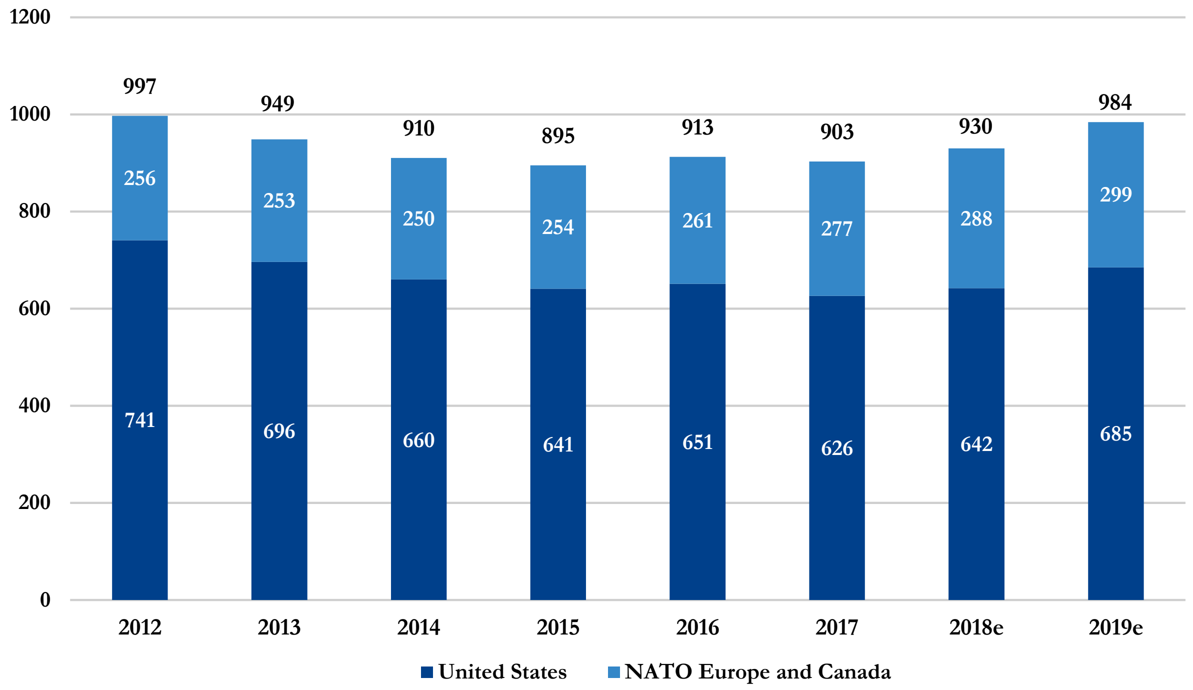

Notes: Figures for 2018 and 2019 are estimates. The NATO Europe and Canada aggregate from 2017 onwards includes Montenegro, which became an Ally on 5 June 2017.

Graph 8 : Defence expenditure as a share of GDP (%)

(based on 2015 prices and exchange rates)

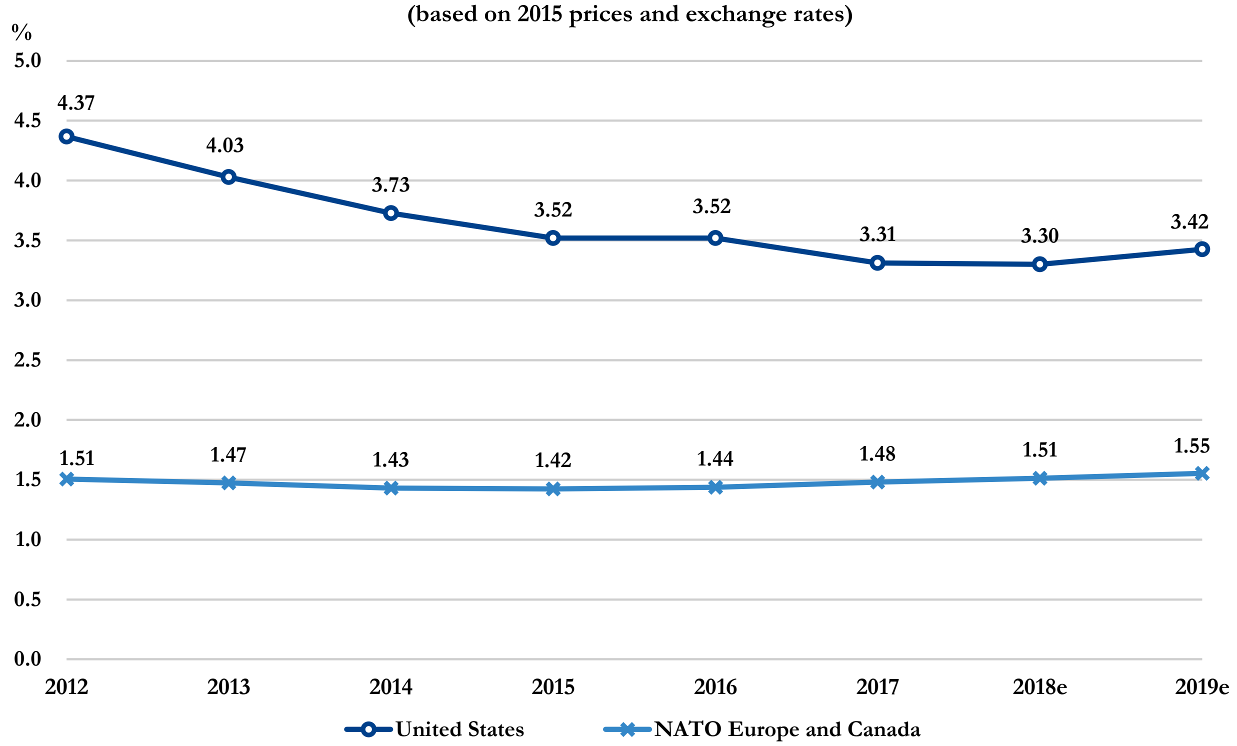

Notes: Figures for 2018 and 2019 are estimates. The NATO Europe and Canada aggregate from 2017 onwards includes Montenegro, which became an Ally on 5 June 2017.

Table 1 : Defence expenditure

Million national currency units

	2012	2013	2014	2015	2016	2017	2018e	2019e
**Current prices**								
Albania (Leks)	19,820	19,022	18,788	16,671	16,250	17,199	18,995	21,702
Belgium (Euros)	4,023	3,964	3,913	3,789	3,848	3,932	4,101	4,303
Bulgaria* (Leva)	1,099	1,196	1,102	1,116	1,186	1,255	1,593	1,840
Canada (Canadian dollars)	19,978	18,764	20,076	23,900	23,474	30,761	28,595	28,810
Croatia (Kunas)	5,059	4,848	6,113	6,057	5,696	6,120	6,560	6,943
Czech Republic (Koruny)	42,780	42,035	41,003	47,264	45,598	52,714	59,671	66,777
Denmark (Kroner)	25,618	23,682	22,769	22,633	24,190	24,961	28,787	31,100
Estonia (Euros)	340	361	386	418	450	479	514	585
France (Euros)	39,105	39,402	39,149	39,199	39,950	40,852	42,748	44,300
Germany (Euros)	36,168	34,593	34,749	35,898	37,598	40,447	41,913	47,320
Greece (Euros)	4,384	3,999	3,939	4,073	4,190	4,213	4,111	4,230
Hungary (Forint)	297,650	286,341	281,402	316,338	362,798	402,793	484,031	553,383
Italy (Euros)	20,600	20,078	18,427	17,642	20,226	21,166	21,183	21,408
Latvia** (Euros)	193	212	221	254	364	470	594	633
Lithuania** (Euros)	252	267	322	425	575	724	895	948
Luxembourg (Euros)	167	176	190	225	213	288	316	342
Montenegro (Euros)	53	49	52	51	56	59	71	80
Netherlands (Euros)	8,067	7,702	7,788	7,816	8,234	8,539	9,417	10,860
Norway (Kroner)	41,560	43,518	46,234	46,900	50,937	53,460	57,473	61,581
Poland** (Zlotys)	28,365	28,467	31,874	39,940	37,082	37,558	42,821	45,379
Portugal (Euros)	2,366	2,457	2,263	2,384	2,364	2,398	2,728	2,928
Romania** (New Lei)	7,282	8,160	9,014	10,337	10,738	14,765	17,183	20,902
Slovak Republic (Euros)	794	729	752	889	907	935	1,098	1,666
Slovenia (Euros)	423	382	366	361	406	422	466	508
Spain (Euros)	10,828	9,495	9,508	10,000	9,014	10,528	11,171	11,505
Turkey (Liras)	24,956	27,466	29,727	32,522	38,203	47,323	68,300	79,356
United Kingdom (Pounds)	36,563	39,824	39,902	38,940	41,590	43,257	45,309	46,564
United States (US dollars)	712,947	680,856	653,942	641,253	656,059	642,936	672,255	730,149
**Constant 2015 prices**								
Albania (Leks)	20,299	19,426	18,894	16,671	16,354	17,057	18,700	20,921
Belgium (Euros)	4,135	4,032	3,952	3,789	3,780	3,799	3,918	4,052
Bulgaria* (Leva)	1,121	1,228	1,126	1,116	1,160	1,187	1,454	1,635
Canada (Canadian dollars)	20,533	18,956	19,894	23,900	23,292	29,769	27,214	27,215
Croatia (Kunas)	5,101	4,850	6,115	6,057	5,700	6,058	6,382	6,655
Czech Republic (Koruny)	44,987	43,580	41,481	47,264	45,029	51,319	56,876	61,817
Denmark (Kroner)	26,225	24,030	22,868	22,633	24,017	24,438	28,065	29,834
Estonia (Euros)	366	376	390	418	443	454	466	513
France (Euros)	40,087	40,081	39,595	39,199	39,873	40,506	41,993	43,014
Germany (Euros)	38,269	35,898	35,439	35,898	37,092	39,298	39,980	44,300
Greece (Euros)	4,176	3,908	3,925	4,073	4,195	4,198	4,077	4,170
Hungary (Forint)	322,946	302,053	286,921	316,338	359,267	385,004	442,784	485,889
Italy (Euros)	21,886	20,771	18,734	17,642	19,769	20,553	20,791	20,992
Latvia** (Euros)	200	216	221	254	361	452	547	561
Lithuania** (Euros)	259	271	323	425	567	685	818	842
Luxembourg (Euros)	174	180	190	225	211	280	295	311
Montenegro (Euros)	56	51	53	51	54	54	64	70
Netherlands (Euros)	8,275	7,780	7,796	7,816	8,162	8,340	9,003	10,243
Norway (Kroner)	44,995	45,702	47,678	46,900	49,974	51,190	54,132	56,562
Poland** (Zlotys)	28,810	28,829	32,119	39,940	36,967	36,723	41,350	42,882
Portugal (Euros)	2,488	2,525	2,309	2,384	2,323	2,321	2,604	2,762
Romania** (New Lei)	7,860	8,519	9,249	10,337	10,481	13,770	15,136	17,496
Slovak Republic (Euros)	796	727	750	889	911	928	1,068	1,581
Slovenia (Euros)	438	389	370	361	403	413	445	474
Spain (Euros)	10,900	9,525	9,557	10,000	8,988	10,369	10,894	11,123
Turkey (Liras)	30,719	31,814	32,054	32,522	35,341	39,495	49,074	48,959
United Kingdom (Pounds)	38,048	40,682	40,076	38,940	40,750	41,470	42,628	42,890
United States (US dollars)	741,064	696,291	660,062	641,253	651,201	626,380	642,012	685,099

Notes: Figures for 2018 and 2019 are estimates.

* Defence expenditure does not include pensions.

** These Allies have national laws and political agreements which call for 2% of GDP to be spent on defence annually, consequently estimates are expected to change accordingly. For the past years, Allies' defence spending was based on the then available GDP data and Allies may, therefore, have met the 2% guideline when using those figures (In 2018, Lithuania met 2% using November 2018 OECD figures).

Table 2 : Defence expenditure

Million US dollars

	2012	2013	2014	2015	2016	2017	2018e	2019e
**Current prices and exchange rates**								
**NATO Europe**	**264,517**	**269,434**	**270,430**	**235,336**	**237,267**	**250,624**	**275,970**	**284,043**
Albania	183	180	178	132	131	144	176	198
Belgium	5,169	5,264	5,192	4,202	4,256	4,431	4,840	4,921
Bulgaria*	722	811	747	633	671	723	961	1,079
Croatia	865	850	1,064	883	837	924	1,045	1,072
Czech Republic	2,185	2,148	1,975	1,921	1,866	2,255	2,746	2,969
Denmark	4,423	4,217	4,057	3,364	3,593	3,780	4,559	4,760
Estonia	437	480	513	463	497	540	607	669
France	50,245	52,316	51,940	43,474	44,191	46,036	50,459	50,659
Germany	46,470	45,931	46,102	39,813	41,590	45,580	49,473	54,113
Greece	5,633	5,309	5,226	4,517	4,635	4,748	4,853	4,844
Hungary	1,322	1,280	1,210	1,132	1,289	1,468	1,791	2,080
Italy	26,468	26,658	24,448	19,566	22,373	23,852	25,004	24,482
Latvia**	248	281	293	281	403	530	701	724
Lithuania**	324	355	427	471	636	816	1,056	1,084
Luxembourg	214	234	253	249	236	325	373	391
Montenegro	68	65	69	57	62	66	84	92
Netherlands	10,365	10,226	10,332	8,668	9,108	9,622	11,115	12,419
Norway	7,143	7,407	7,337	5,816	6,064	6,463	7,067	7,179
Poland**	9,574	9,007	10,104	10,596	9,405	9,938	11,856	11,971
Portugal	3,040	3,262	3,003	2,644	2,615	2,702	3,220	3,358
Romania**	2,100	2,452	2,691	2,581	2,645	3,643	4,359	5,043
Slovak Republic	1,020	968	997	986	1,003	1,053	1,297	1,905
Slovenia	543	507	486	401	449	476	550	581
Spain	13,912	12,607	12,614	11,090	9,971	11,864	13,186	13,156
Turkey	13,895	14,427	13,583	11,957	12,649	12,972	14,145	13,919
United Kingdom	58,016	62,258	65,658	59,492	56,154	55,672	60,446	60,376
**North America**	**732,941**	**699,077**	**672,092**	**659,938**	**673,770**	**666,640**	**694,323**	**752,034**
Canada	19,994	18,221	18,150	18,685	17,711	23,704	22,068	21,885
United States	712,947	680,856	653,942	641,253	656,059	642,936	672,255	730,149
**NATO Total**	**997,459**	**968,512**	**942,522**	**895,274**	**911,037**	**917,263**	**970,293**	**1,036,077**
**Constant 2015 prices and exchange rates**								
**NATO Europe**	**239,924**	**237,769**	**234,571**	**235,336**	**243,114**	**253,307**	**266,502**	**277,858**
Albania	161	154	150	132	130	135	148	166
Belgium	4,586	4,472	4,383	4,202	4,192	4,214	4,345	4,494
Bulgaria*	635	696	638	633	657	673	824	927
Croatia	744	707	892	883	831	883	931	970
Czech Republic	1,829	1,772	1,686	1,921	1,831	2,086	2,312	2,513
Denmark	3,898	3,572	3,399	3,364	3,570	3,632	4,171	4,434
Estonia	406	417	432	463	491	504	517	569
France	44,460	44,453	43,914	43,474	44,222	44,923	46,573	47,705
Germany	42,443	39,813	39,304	39,813	41,138	43,584	44,341	49,132
Greece	4,632	4,335	4,353	4,517	4,652	4,655	4,522	4,624
Hungary	1,156	1,081	1,027	1,132	1,286	1,378	1,585	1,739
Italy	24,273	23,036	20,777	19,566	21,925	22,794	23,058	23,281
Latvia**	221	239	245	281	400	501	607	622
Lithuania**	287	301	358	471	629	759	907	933
Luxembourg	193	200	210	249	234	310	328	345
Montenegro	62	56	59	57	59	60	71	78
Netherlands	9,178	8,629	8,646	8,668	9,053	9,249	9,985	11,360
Norway	5,580	5,667	5,912	5,816	6,197	6,348	6,713	7,014
Poland**	7,643	7,648	8,521	10,596	9,807	9,742	10,970	11,376
Portugal	2,759	2,801	2,561	2,644	2,576	2,574	2,888	3,064
Romania**	1,962	2,127	2,309	2,581	2,617	3,438	3,779	4,368
Slovak Republic	882	806	832	986	1,011	1,029	1,184	1,754
Slovenia	485	431	411	401	447	458	494	525
Spain	12,089	10,564	10,599	11,090	9,968	11,500	12,082	12,336
Turkey	11,294	11,696	11,784	11,957	12,993	14,520	18,042	18,000
United Kingdom	58,129	62,153	61,227	59,492	62,257	63,357	65,126	65,527
**North America**	**757,116**	**711,111**	**675,615**	**659,938**	**669,411**	**649,653**	**663,288**	**706,376**
Canada	16,053	14,820	15,553	18,685	18,210	23,273	21,276	21,277
United States	741,064	696,291	660,062	641,253	651,201	626,380	642,012	685,099
**NATO Total**	**997,040**	**948,880**	**910,187**	**895,274**	**912,524**	**902,961**	**929,790**	**984,234**

Notes: Figures for 2018 and 2019 are estimates. The NATO Europe and NATO Total aggregates from 2017 onwards include Montenegro, which became an Ally on 5 June 2017.

* Defence expenditure does not include pensions.

** These Allies have national laws and political agreements which call for 2% of GDP to be spent on defence annually, consequently estimates are expected to change accordingly. For the past years, Allies' defence spending was based on the then available GDP data and Allies may, therefore, have met the 2% guideline when using those figures (In 2018, Lithuania met 2% using November 2018 OECD figures).

Table 3 : Defence expenditure as a share of GDP and annual real change

Based on 2015 prices

	2012	2013	2014	2015	2016	2017	2018e	2019e
**Share of real GDP (%)**								
**NATO Europe**	**1.55**	**1.52**	**1.47**	**1.45**	**1.46**	**1.48**	**1.53**	**1.58**
Albania	1.49	1.41	1.35	1.16	1.10	1.11	1.17	1.26
Belgium	1.04	1.01	0.98	0.92	0.91	0.90	0.91	0.93
Bulgaria*	1.34	1.46	1.32	1.26	1.26	1.24	1.48	1.61
Croatia	1.53	1.46	1.84	1.78	1.62	1.67	1.72	1.75
Czech Republic	1.05	1.03	0.95	1.03	0.96	1.04	1.12	1.19
Denmark	1.35	1.23	1.15	1.11	1.15	1.15	1.30	1.35
Estonia	1.90	1.91	1.93	2.02	2.07	2.03	2.00	2.13
France	1.87	1.86	1.82	1.78	1.79	1.78	1.82	1.84
Germany	1.31	1.22	1.18	1.18	1.19	1.23	1.24	1.36
Greece	2.29	2.21	2.21	2.30	2.38	2.34	2.23	2.24
Hungary	1.03	0.95	0.86	0.92	1.02	1.05	1.15	1.21
Italy	1.32	1.27	1.14	1.07	1.18	1.21	1.21	1.22
Latvia**	0.88	0.93	0.94	1.04	1.45	1.74	2.01	2.01
Lithuania**	0.76	0.76	0.88	1.14	1.48	1.72	1.98	1.98
Luxembourg	0.38	0.38	0.38	0.44	0.40	0.52	0.54	0.55
Montenegro	1.66	1.47	1.50	1.40	1.42	1.36	1.54	1.65
Netherlands	1.24	1.17	1.15	1.13	1.16	1.15	1.21	1.35
Norway	1.52	1.52	1.56	1.50	1.58	1.59	1.66	1.70
Poland**	1.74	1.72	1.85	2.22	1.99	1.89	2.02	2.01
Portugal	1.41	1.44	1.31	1.33	1.27	1.23	1.35	1.41
Romania**	1.23	1.28	1.35	1.45	1.40	1.72	1.82	2.04
Slovak Republic	1.09	0.98	0.99	1.12	1.12	1.10	1.22	1.74
Slovenia	1.17	1.05	0.97	0.93	1.01	0.98	1.01	1.04
Spain	1.04	0.93	0.92	0.92	0.81	0.90	0.92	0.92
Turkey	1.59	1.52	1.45	1.39	1.46	1.52	1.85	1.89
United Kingdom	2.16	2.26	2.16	2.05	2.11	2.11	2.14	2.13
**North America**	**4.11**	**3.78**	**3.51**	**3.34**	**3.33**	**3.16**	**3.14**	**3.26**
Canada	1.09	0.99	1.01	1.20	1.16	1.44	1.29	1.27
United States	4.37	4.03	3.73	3.52	3.52	3.31	3.30	3.42
**NATO Total**	**2.94**	**2.76**	**2.59**	**2.48**	**2.49**	**2.40**	**2.42**	**2.51**
**Annual real change (%)**								
**NATO Europe**	**-2.22**	**-0.90**	**-1.34**	**0.33**	**3.31**	**4.19**	**5.21**	**4.26**
Albania	-1.32	-4.30	-2.74	-11.76	-1.90	4.30	9.64	11.88
Belgium	-0.27	-2.48	-1.99	-4.13	-0.25	0.52	3.12	3.43
Bulgaria*	1.54	9.53	-8.29	-0.84	3.88	2.33	22.52	12.48
Croatia	-6.41	-4.91	26.07	-0.94	-5.90	6.28	5.35	4.28
Czech Republic	-2.24	-3.13	-4.82	13.94	-4.73	13.97	10.83	8.69
Denmark	3.15	-8.37	-4.84	-1.03	6.12	1.75	14.84	6.30
Estonia	17.71	2.72	3.75	7.12	6.11	2.55	2.57	9.98
France	0.55	-0.02	-1.21	-1.00	1.72	1.59	3.67	2.43
Germany	2.86	-6.20	-1.28	1.30	3.33	5.95	1.74	10.81
Greece	-10.79	-6.41	0.42	3.78	2.99	0.06	-2.87	2.27
Hungary	-2.59	-6.47	-5.01	10.25	13.57	7.16	15.01	9.74
Italy	-6.82	-5.09	-9.81	-5.83	12.05	3.97	1.16	0.97
Latvia**	-9.53	7.94	2.55	14.79	42.29	25.14	21.09	2.51
Lithuania**	-0.90	4.72	19.15	31.60	33.37	20.79	19.51	2.88
Luxembourg	-2.29	3.70	5.33	18.59	-6.04	32.39	5.59	5.24
Montenegro	-8.11	-8.44	4.49	-3.50	4.33	0.28	18.72	10.39
Netherlands	-2.84	-5.98	0.19	0.26	4.43	2.17	7.96	13.77
Norway	0.40	1.57	4.32	-1.63	6.55	2.43	5.75	4.49
Poland**	2.73	0.07	11.41	24.35	-7.44	-0.66	12.60	3.70
Portugal	-9.56	1.51	-8.56	3.24	-2.55	-0.08	12.17	6.10
Romania**	-3.50	8.39	8.57	11.76	1.39	31.38	9.92	15.59
Slovak Republic	2.31	-8.63	3.22	18.54	2.47	1.78	15.09	48.11
Slovenia	-12.07	-11.19	-4.74	-2.39	11.55	2.34	7.88	6.45
Spain	7.57	-12.62	0.33	4.64	-10.12	15.37	5.07	2.10
Turkey	1.87	3.56	0.75	1.46	8.67	11.75	24.25	-0.24
United Kingdom	-8.17	6.92	-1.49	-2.83	4.65	1.77	2.79	0.61
**North America**	**-5.54**	**-6.08**	**-4.99**	**-2.32**	**1.44**	**-2.95**	**2.10**	**6.50**
Canada	-9.49	-7.68	4.95	20.14	-2.54	27.81	-8.58	0.00
United States	-5.45	-6.04	-5.20	-2.85	1.55	-3.81	2.50	6.71
**NATO Total**	**-4.76**	**-4.83**	**-4.08**	**-1.64**	**1.93**	**-1.05**	**2.97**	**5.86**

Notes: Figures for 2018 and 2019 are estimates. The NATO Europe and NATO Total aggregates from 2017 onwards include Montenegro, which became an Ally on 5 June 2017.

* Defence expenditure does not include pensions.

** These Allies have national laws and political agreements which call for 2% of GDP to be spent on defence annually, consequently estimates are expected to change accordingly. For the past years, Allies' defence spending was based on the then available GDP data and Allies may, therefore, have met the 2% guideline when using those figures (In 2018, Lithuania met 2% using November 2018 OECD figures).

Table 4 : Defence expenditure real change 2014-2019

Million US dollars (2015 prices and exchange rates)

	2014	2019e	Real change 2014-2019e (%)	Share of real GDP 2014 (%)	Share of real GDP 2019e (%)
Albania	150	166	10.73	1.35	1.26
Belgium	4,383	4,494	2.53	0.98	0.93
Bulgaria*	638	927	45.26	1.32	1.61
Canada	15,553	21,277	36.80	1.01	1.27
Croatia	892	970	8.84	1.84	1.75
Czech Republic	1,686	2,513	49.02	0.95	1.19
Denmark	3,399	4,434	30.46	1.15	1.35
Estonia	432	569	31.48	1.93	2.13
France	43,914	47,705	8.63	1.82	1.84
Germany	39,304	49,132	25.01	1.18	1.36
Greece	4,353	4,624	6.23	2.21	2.24
Hungary	1,027	1,739	69.35	0.86	1.21
Italy	20,777	23,281	12.05	1.14	1.22
Latvia**	245	622	153.70	0.94	2.01
Lithuania**	358	933	160.65	0.88	1.98
Luxembourg	210	345	63.93	0.38	0.55
Montenegro	59	78	32.32	1.50	1.65
Netherlands	8,646	11,360	31.40	1.15	1.35
Norway	5,912	7,014	18.63	1.56	1.70
Poland**	8,521	11,376	33.51	1.85	2.01
Portugal	2,561	3,064	19.63	1.31	1.41
Romania**	2,309	4,368	89.16	1.35	2.04
Slovak Republic	832	1,754	110.74	0.99	1.74
Slovenia	411	525	27.98	0.97	1.04
Spain	10,599	12,336	16.39	0.92	0.92
Turkey	11,784	18,000	52.74	1.45	1.89
United Kingdom	61,227	65,527	7.02	2.16	2.13
United States	660,062	685,099	3.79	3.73	3.42

Notes: Figures for 2018 and 2019 are estimates.

* Defence expenditure does not include pensions.

** These Allies have national laws and political agreements which call for 2% of GDP to be spent on defence annually, consequently estimates are expected to change accordingly. For the past years, Allies' defence spending was based on the then available GDP data and Allies may, therefore, have met the 2% guideline when using those figures (In 2018, Lithuania met 2% using November 2018 OECD figures).

Table 5 : Real GDP

Billion US dollars (2015 prices and exchange rates)

	2012	2013	2014	2015	2016	2017	2018e	2019e
**NATO Europe**	**15,526**	**15,640**	**15,945**	**16,282**	**16,603**	**17,058**	**17,372**	**17,568**
Albania	11	11	11	11	12	12	13	13
Belgium	442	443	448	456	463	471	477	483
Bulgaria	47	48	49	50	52	54	56	58
Croatia	49	48	48	50	51	53	54	56
Czech Republic	174	173	177	187	191	200	206	211
Denmark	288	291	296	303	310	317	321	328
Estonia	21	22	22	23	24	25	26	27
France	2,375	2,390	2,413	2,438	2,465	2,522	2,561	2,595
Germany	3,238	3,258	3,329	3,378	3,451	3,536	3,587	3,614
Greece	202	196	197	196	196	199	202	207
Hungary	112	114	119	123	126	131	138	143
Iceland	16	16	17	17	19	19	20	20
Italy	1,845	1,813	1,816	1,831	1,854	1,886	1,900	1,901
Latvia*	25	26	26	27	28	29	30	31
Lithuania*	38	39	41	42	42	44	46	47
Luxembourg	51	53	55	57	59	59	61	62
Montenegro	4	4	4	4	4	4	5	5
Netherlands	741	740	751	765	782	805	826	840
Norway	368	372	379	387	391	399	405	412
Poland*	439	445	460	478	492	516	542	565
Portugal	196	194	196	199	203	209	213	217
Romania*	160	166	171	178	186	199	208	214
Slovak Republic	81	82	84	88	91	93	97	101
Slovenia	41	41	42	43	44	47	49	50
Spain	1,161	1,141	1,157	1,199	1,237	1,274	1,307	1,336
Turkey	710	771	810	860	887	953	978	952
United Kingdom	2,694	2,749	2,830	2,896	2,948	3,002	3,044	3,081
**North America**	**18,441**	**18,788**	**19,255**	**19,775**	**20,078**	**20,535**	**21,105**	**21,675**
Canada	1,468	1,502	1,545	1,556	1,573	1,620	1,650	1,671
United States	16,973	17,285	17,709	18,219	18,505	18,915	19,456	20,004
**NATO Total**	**33,967**	**34,428**	**35,200**	**36,058**	**36,682**	**37,594**	**38,477**	**39,243**

Notes: Figures for 2018 and 2019 are estimates. The NATO Europe and NATO Total aggregates from 2017 onwards include Montenegro, which became an Ally on 5 June 2017.

* These Allies have national laws and political agreements which call for 2% of GDP to be spent on defence annually, consequently estimates are expected to change accordingly. For the past years, Allies' defence spending was based on the then available GDP data and Allies may, therefore, have met the 2% guideline when using those figures (In 2018, Lithuania met 2% using November 2018 OECD figures).

Table 6 : GDP per capita and defence expenditure per capita

2015 prices and exchange rates

	2012	2013	2014	2015	2016	2017	2018e	2019e
**GDP per capita (thousand US dollars)**								
**NATO Europe**	**27.7**	**27.8**	**28.3**	**28.8**	**29.2**	**29.9**	**30.4**	**30.6**
Albania	3.7	3.8	3.9	4.0	4.1	4.3	4.4	4.6
Belgium	39.9	39.8	40.1	40.6	41.0	41.5	41.9	42.1
Bulgaria	6.5	6.6	6.7	7.0	7.3	7.7	7.9	8.2
Croatia	11.4	11.4	11.4	11.8	12.3	12.8	13.2	13.6
Czech Republic	16.5	16.4	16.9	17.7	18.1	18.9	19.3	19.8
Denmark	51.6	51.8	52.4	53.3	54.1	55.0	55.5	56.2
Estonia	16.1	16.5	17.1	17.4	18.0	18.9	19.6	20.1
France	36.2	36.2	36.4	36.6	36.9	37.7	38.2	38.6
Germany	40.3	40.4	41.1	41.4	41.9	42.8	43.3	43.4
Greece	18.3	17.8	18.1	18.1	18.2	18.5	18.9	19.3
Hungary	11.3	11.5	12.1	12.5	12.8	13.4	14.1	14.7
Iceland	48.7	50.2	50.7	52.6	55.3	56.5	58.6	58.7
Italy	30.6	29.9	29.9	30.1	30.6	31.2	31.4	31.4
Latvia	12.3	12.8	13.1	13.6	14.1	14.8	15.7	16.2
Lithuania	12.7	13.3	13.9	14.3	14.8	15.7	16.3	16.9
Luxembourg	95.7	96.7	98.5	100.4	100.2	99.6	99.9	99.7
Montenegro	6.0	6.2	6.3	6.5	6.7	7.1	7.4	7.6
Netherlands	44.2	44.0	44.5	45.2	45.9	47.0	47.9	48.4
Norway	73.3	73.2	73.8	74.5	74.7	75.6	76.1	76.8
Poland	11.4	11.6	12.0	12.4	12.8	13.4	14.1	14.7
Portugal	18.7	18.6	18.8	19.3	19.7	20.3	20.8	21.2
Romania	8.0	8.3	8.6	9.0	9.5	10.2	10.6	11.0
Slovak Republic	14.9	15.1	15.5	16.2	16.7	17.2	17.9	18.4
Slovenia	20.1	19.9	20.4	20.9	21.5	22.6	23.5	24.3
Spain	24.8	24.5	24.9	25.8	26.6	27.4	28.0	28.5
Turkey	9.5	10.2	10.6	11.1	11.3	12.1	12.2	11.7
United Kingdom	42.3	42.9	43.8	44.5	44.9	45.5	45.8	46.0
**North America**	**52.8**	**53.4**	**54.3**	**55.4**	**55.8**	**56.6**	**57.8**	**58.9**
Canada	42.2	42.7	43.5	43.4	43.4	44.1	44.5	44.7
United States	54.0	54.6	55.5	56.7	57.2	58.0	59.3	60.5
**NATO Total**	**37.4**	**37.7**	**38.3**	**39.1**	**39.5**	**40.3**	**41.0**	**41.6**
**Defence expenditure per capita (US dollars)**								
**NATO Europe**	**430**	**423**	**417**	**422**	**433**	**456**	**469**	**486**
Albania	56	53	52	46	45	47	52	58
Belgium	414	402	392	374	371	371	381	392
Bulgaria*	87	96	88	88	92	95	117	132
Croatia	174	166	210	210	199	214	227	238
Czech Republic	174	169	160	182	173	197	217	236
Denmark	697	636	602	592	623	630	720	760
Estonia	306	316	329	353	373	383	392	429
France	677	674	662	653	663	672	695	709
Germany	528	494	485	487	500	527	535	591
Greece	419	395	400	417	432	433	421	431
Hungary	117	109	104	115	131	141	162	178
Italy	402	380	342	322	362	377	381	385
Latvia**	109	119	123	142	204	258	315	325
Lithuania**	96	102	122	162	219	269	324	336
Luxembourg	362	366	377	438	401	520	537	552
Montenegro	99	91	95	91	95	96	114	126
Netherlands	548	514	513	512	532	540	579	655
Norway	1,112	1,116	1,151	1,120	1,184	1,203	1,263	1,308
Poland**	198	199	221	276	255	254	286	296
Portugal	262	268	246	255	250	250	281	299
Romania**	98	106	116	130	133	175	193	225
Slovak Republic	163	149	154	182	186	189	217	322
Slovenia	236	209	199	194	217	221	238	253
Spain	259	227	228	239	215	247	258	264
Turkey	151	154	154	154	166	184	225	222
United Kingdom	912	970	948	914	948	959	980	979
**North America**	**2,169**	**2,022**	**1,906**	**1,848**	**1,860**	**1,791**	**1,816**	**1,919**
Canada	462	422	438	521	502	634	574	569
United States	2,357	2,199	2,069	1,996	2,012	1,922	1,956	2,072
**NATO Total**	**1,097**	**1,039**	**991**	**970**	**984**	**968**	**992**	**1,045**

Notes: Figures for 2018 and 2019 are estimates. The NATO Europe and NATO Total aggregates from 2017 onwards include Montenegro, which became an Ally on 5 June 2017.

* Defence expenditure does not include pensions.

** These Allies have national laws and political agreements which call for 2% of GDP to be spent on defence annually, consequently estimates are expected to change accordingly. For the past years, Allies' defence spending was based on the then available GDP data and Allies may, therefore, have met the 2% guideline when using those figures (In 2018, Lithuania met 2% using November 2018 OECD figures).

Table 7 : Military personnel

Thousands

	2012	2013	2014	2015	2016	2017	2018e	2019e
**NATO Europe**	**1,956**	**1,862**	**1,825**	**1,740**	**1,718**	**1,775**	**1,845**	**1,849**
Albania	7.9	6.8	6.7	6.2	5.8	6.8	6.8	6.8
Belgium	31	31	31	30	29	28	27	26
Bulgaria	26	28	27	25	25	25	25	25
Croatia	16	16	15	15	15	15	15	15
Czech Republic	22	20	20	21	23	24	25	26
Denmark	19	18	17	17	17	17	17	17
Estonia	6.0	6.3	6.3	6.0	6.1	6.2	6.2	6.3
France	219	213	207	205	208	208	208	208
Germany	192	184	179	177	178	180	183	184
Greece	110	110	107	104	106	106	105	105
Hungary	19	18	17	17	18	19	19	20
Italy	189	189	183	178	176	175	180	179
Latvia	4.7	4.6	4.6	4.8	5.2	5.5	6.3	6.4
Lithuania	8.3	8.4	8.6	11.8	11.8	13.5	14.3	15.9
Luxembourg	0.8	0.9	0.8	0.8	0.8	0.8	0.9	0.9
Montenegro	1.9	1.9	1.9	1.7	1.5	1.5	1.5	1.6
Netherlands	44	42	41	41	40	39	41	41
Norway	21	20	21	21	20	20	20	20
Poland	98	100	99	99	102	105	118	123
Portugal	34	33	31	28	30	28	30	30
Romania	66	66	65	64	63	62	69	69
Slovak Republic	13	12	12	12	12	12	13	13
Slovenia	7.1	6.9	6.8	6.6	6.5	6.3	6.8	6.8
Spain	125	122	122	122	121	118	117	121
Turkey	495	427	427	385	359	417	444	435
United Kingdom	184	179	169	141	139	137	145	144
**North America**	**1,467**	**1,450**	**1,404**	**1,384**	**1,372**	**1,379**	**1,394**	**1,410**
Canada	68	68	66	70	71	71	71	72
United States	1,400	1,382	1,338	1,314	1,301	1,307	1,323	1,338
**NATO Total**	**3,423**	**3,312**	**3,229**	**3,125**	**3,090**	**3,153**	**3,239**	**3,258**

Notes: Figures for 2018 and 2019 are estimates. The NATO Europe and NATO Total aggregates from 2017 onwards include Montenegro, which became an Ally on 5 June 2017.

Table 8a : Distribution of defence expenditure by main category

Percentage of total defence expenditure

	2012	2013	2014	2015	2016	2017	2018e	2019e
**Equipment (a)**								
Albania	14.44	16.29	16.65	8.92	8.01	6.96	9.42	14.42
Belgium	3.57	2.84	3.52	3.44	4.72	6.52	10.15	10.78
Bulgaria*	3.68	4.52	1.03	3.47	9.15	8.10	9.65	25.13
Canada	8.31	11.16	13.03	10.47	10.61	10.70	12.58	11.25
Croatia	14.72	10.72	5.56	8.01	7.51	5.69	9.45	11.64
Czech Republic	14.78	9.49	6.53	11.76	6.70	11.12	10.57	14.50
Denmark	9.03	11.26	10.99	11.50	13.68	10.39	11.66	17.04
Estonia	13.69	14.48	22.15	12.82	17.86	19.22	16.96	19.37
France	30.58	28.56	24.64	25.04	24.44	24.17	23.66	24.44
Germany	16.45	12.74	12.94	11.93	12.21	13.75	14.13	16.40
Greece	7.47	12.06	8.17	10.40	13.45	15.47	12.40	12.53
Hungary	5.84	11.08	7.76	9.75	13.37	15.34	20.35	23.48
Italy	8.87	12.51	10.92	9.72	19.09	20.68	21.12	20.57
Latvia	10.45	12.09	7.55	13.60	19.05	17.22	28.06	25.29
Lithuania	11.20	9.23	14.06	21.55	30.06	31.61	36.98	28.68
Luxembourg	17.11	14.57	22.61	33.33	30.07	42.06	41.77	44.62
Montenegro	4.44	1.32	7.46	5.43	4.46	4.89	9.66	14.26
Netherlands	13.41	12.57	10.68	11.16	14.14	14.75	16.46	23.09
Norway	17.76	18.89	21.17	22.49	24.05	24.70	24.67	29.51
Poland	15.16	13.90	18.84	33.20	21.62	22.04	27.49	23.87
Portugal	9.34	8.65	8.43	8.70	9.95	10.02	12.97	11.03
Romania	4.14	10.71	15.77	19.65	20.43	33.34	33.47	24.80
Slovak Republic	9.56	7.39	11.12	18.28	15.32	17.74	22.27	41.68
Slovenia	1.20	1.27	0.66	1.85	1.02	4.04	8.22	7.96
Spain	22.86	12.37	13.49	14.82	6.65	20.39	21.82	20.72
Turkey	21.21	26.89	25.08	25.13	25.55	30.30	37.64	38.60
United Kingdom	19.54	21.89	22.82	21.75	21.24	22.29	24.23	24.11
United States	26.97	25.83	25.97	25.41	25.05	25.73	27.06	27.51
**Personnel (b)**								
Albania	69.97	75.25	68.05	78.15	68.05	68.20	70.70	64.78
Belgium	78.52	77.34	77.84	78.23	76.80	75.20	70.69	68.65
Bulgaria*	64.71	65.37	72.84	73.66	65.64	68.33	62.99	56.19
Canada	49.10	52.44	50.90	53.76	53.11	57.37	50.73	49.74
Croatia	68.13	68.06	76.55	72.28	75.40	71.72	71.30	68.49
Czech Republic	61.66	62.03	61.40	55.27	61.97	56.19	54.63	..
Denmark	49.05	51.74	51.27	52.01	49.51	47.01	49.88	..
Estonia	29.75	39.83	38.62	39.56	38.70	34.89	33.65	34.33
France	49.11	49.23	48.59	47.79	47.94	47.98	46.90	..
Germany	50.60	49.86	50.67	49.86	48.35	48.75	46.97	..
Greece	73.19	74.56	77.18	72.05	73.13	72.19	71.36	69.56
Hungary	47.69	48.96	49.77	48.21	49.66	42.41	39.98	41.72
Italy	77.05	75.00	76.41	77.55	70.79	67.58	65.66	..
Latvia	56.15	52.98	52.97	50.06	43.87	37.26	32.05	35.25
Lithuania	66.78	66.53	57.53	48.49	45.50	40.79	37.47	43.26
Luxembourg	54.23	51.10	49.31	42.77	45.56	34.40	32.79	..
Montenegro	82.68	87.68	78.53	78.03	75.32	79.56	71.65	64.46
Netherlands	57.54	58.53	56.50	55.51	51.77	52.19	51.16	45.28
Norway	42.38	41.02	39.36	38.70	37.28	36.10	35.99	32.80
Poland	57.34	57.70	51.45	41.96	47.15	50.04	46.14	48.26
Portugal	78.39	79.85	81.27	81.90	81.38	81.52	71.79	..
Romania	84.00	78.99	71.15	63.30	65.01	54.67	54.48	56.91
Slovak Republic	66.53	70.14	69.14	56.24	58.72	58.21	54.74	40.61
Slovenia	78.91	80.52	82.31	82.23	76.03	75.05	72.43	..
Spain	57.19	68.25	67.34	65.18	72.61	61.64	59.65	60.80
Turkey	56.02	54.58	56.88	56.82	57.60	51.02	45.18	46.75
United Kingdom	38.90	37.85	36.59	36.80	35.27	34.54	33.79	35.61
United States	32.12	34.38	35.45	36.64	45.01	41.22	39.28	38.62

Notes: Figures for 2018 and 2019 are estimates.

* Defence expenditure does not include pensions.

(a) Equipment expenditure includes major equipment expenditure and R&D devoted to major equipment.

(b) Personnel expenditure includes military and civilian expenditure and pensions.

Table 8b : Distribution of defence expenditure by main category

Percentage of total defence expenditure

	2012	2013	2014	2015	2016	2017	2018e	2019e
**Infrastructure (c)**								
Albania	0.64	1.17	0.86	1.40	1.37	0.92	1.09	1.98
Belgium	1.59	2.28	1.81	0.93	0.96	1.05	1.43	1.30
Bulgaria*	0.76	0.47	0.63	1.27	0.63	0.83	2.62	0.62
Canada	5.47	4.12	3.81	3.63	3.03	2.98	4.20	3.38
Croatia	0.57	1.21	1.24	1.98	1.26	3.59	1.07	2.30
Czech Republic	1.61	2.72	2.34	3.32	3.91	4.00	5.35	..
Denmark	1.24	1.16	0.97	1.09	2.16	1.95	1.49	..
Estonia	8.85	11.54	8.20	8.45	12.15	11.27	8.63	5.63
France	3.42	2.30	2.33	2.80	2.70	2.88	3.51	..
Germany	3.53	3.55	3.75	3.60	3.39	3.91	3.94	..
Greece	0.79	0.63	1.10	0.65	0.58	0.37	0.14	0.14
Hungary	2.11	2.32	1.07	1.21	1.13	1.64	4.85	3.52
Italy	1.02	1.57	1.40	1.30	0.70	0.94	1.92	..
Latvia	4.16	6.26	8.89	6.64	12.83	17.56	7.17	7.64
Lithuania	1.47	2.04	2.17	2.16	3.59	3.92	2.24	5.12
Luxembourg	8.20	11.81	10.26	7.79	6.64	4.64	6.96	4.67
Montenegro	0.03	0.09	0.96	2.47	2.41	0.87	2.68	3.99
Netherlands	3.70	2.74	4.77	3.19	3.90	3.02	3.48	3.21
Norway	4.86	5.64	6.00	5.60	6.96	7.35	7.12	7.64
Poland	4.76	5.62	5.47	4.74	4.62	4.21	3.45	5.32
Portugal	0.04	0.04	0.11	0.25	0.06	0.03	0.37	..
Romania	1.19	1.16	1.09	1.27	2.77	2.09	1.54	4.60
Slovak Republic	0.37	0.29	0.57	1.99	3.75	2.97	2.00	1.90
Slovenia	2.02	1.33	0.65	0.61	1.14	0.45	0.49	..
Spain	0.85	0.67	0.66	0.97	0.97	0.68	0.64	1.10
Turkey	3.70	2.72	2.77	2.56	2.42	2.95	2.53	2.04
United Kingdom	1.91	2.04	1.95	1.63	1.87	2.25	2.98	1.90
United States	2.38	2.08	1.71	1.45	1.22	1.23	1.17	1.27
**Other (d)**								
Albania	14.95	7.30	14.44	11.53	22.57	23.92	18.79	18.81
Belgium	16.31	17.53	16.83	17.40	17.52	17.23	17.72	19.27
Bulgaria*	30.85	29.64	25.51	21.60	24.57	22.74	24.74	18.05
Canada	37.12	32.28	32.26	32.14	33.25	28.95	32.49	35.63
Croatia	16.58	20.01	16.65	17.73	15.83	18.99	18.17	17.57
Czech Republic	21.95	25.75	29.73	29.65	27.43	28.69	29.44	..
Denmark	40.68	35.84	36.78	35.40	34.65	40.66	36.97	..
Estonia	47.71	34.14	31.03	39.18	31.30	34.62	40.76	40.67
France	16.89	19.91	24.43	24.37	24.92	24.97	25.92	..
Germany	29.42	33.84	32.63	34.61	36.05	33.58	34.96	..
Greece	18.55	12.75	13.55	16.90	12.84	11.97	16.10	17.78
Hungary	44.35	37.64	41.40	40.83	35.84	40.62	34.82	31.29
Italy	13.06	10.93	11.27	11.42	9.42	10.80	11.30	..
Latvia	29.23	28.68	30.59	29.69	24.25	27.96	32.72	31.83
Lithuania	20.56	22.20	26.24	27.79	20.85	23.67	23.30	22.94
Luxembourg	20.46	22.52	17.82	16.11	17.73	18.90	18.48	..
Montenegro	12.85	10.91	13.06	14.07	17.80	14.67	16.01	17.29
Netherlands	25.34	26.16	28.05	30.14	30.20	30.04	28.90	28.43
Norway	35.00	34.45	33.46	33.21	31.71	31.84	32.22	30.05
Poland	22.73	22.78	24.24	20.11	26.61	23.71	22.91	22.56
Portugal	12.22	11.46	10.19	9.15	8.61	8.43	14.87	..
Romania	10.68	9.13	11.98	15.78	11.79	9.90	10.51	13.69
Slovak Republic	23.55	22.19	19.16	23.49	22.22	21.08	20.99	15.80
Slovenia	17.87	16.88	16.38	15.31	21.80	20.46	18.87	..
Spain	19.11	18.71	18.50	19.03	19.78	17.28	17.89	17.39
Turkey	19.07	15.80	15.27	15.49	14.43	15.73	14.65	12.61
United Kingdom	39.64	38.22	38.63	39.82	41.62	40.92	38.99	38.38
United States	38.53	37.72	36.87	36.51	28.73	31.83	32.49	32.59

Notes: Figures for 2018 and 2019 are estimates.

* Defence expenditure does not include pensions.

(c) Infrastructure expenditure includes NATO common infrastructure and national military construction.

(d) Other expenditure includes operations and maintenance expenditure, other R&D expenditure and expenditure not allocated among above-mentioned categories.

NATO defence expenditure

NATO defines defence expenditure as payments made by a national government specifically to meet the needs of its armed forces, those of Allies or of the Alliance. A major component of defence expenditure is payments on Armed Forces financed within the Ministry of Defence (MoD) budget. Armed Forces include Land, Maritime and Air forces as well as Joint formations such as Administration and Command, Special Operations Forces, Medical Service, Logistic Command etc. They might also include "Other Forces" like Ministry of Interior troops, national police forces, *gendarmerie*, *carabinieri*, coast guards etc. In such cases, expenditure should be included only in proportion to the forces that are trained in military tactics, are equipped as a military force, can operate under direct military authority in deployed operations, and can, realistically, be deployed outside national territory in support of a military force. Also, expenditure on Other Forces financed through the budgets of ministries other than MoD should be included in defence expenditure.

Pension payments made directly by the government to retired military and civilian employees of military departments should be included regardless of whether these payments are made from the budget of the MoD or other ministries.

Expenditure for peacekeeping and humanitarian operations (paid by MoD or other ministries), the destruction of weapons, equipment and ammunition, contributions to eligible NATO-managed trust funds, and the costs associated with inspection and control of equipment destruction are included in defence expenditure.

Research and development (R&D) costs are to be included in defence expenditure. R&D costs should also include expenditure for those projects that do not successfully lead to production of equipment.

Expenditure for the military component of mixed civilian-military activities is included, but only when the military component can be specifically accounted for or estimated.

Expenditure on NATO Common infrastructure is included in the total defence expenditure of each Ally only to the extent of that nation's net contribution.

War damage payments and spending on civil defence are both excluded from the NATO definition of defence expenditure.

NATO uses United States dollars (USD) as the common currency denominator. The exchange rate applied to each Ally is the average annual rate published by the International Monetary Fund (IMF). The values for defence expenditure are expressed in current prices; constant prices; current prices and exchange rates; as well as constant prices and exchange rates.

Note to readers:

Iceland has no armed forces. For nations of the Euro zone, and Montenegro, monetary values in national currency are expressed in Euros for all years. Estonia adopted Euros from 2011, Latvia from 2014, and Lithuania from 2015. Montenegro joined the Alliance in 2017.

To avoid any ambiguity, the fiscal year has been designated by the year which includes the highest number of months: e.g. 2018 represents the fiscal year 2018/2019 for Canada and United Kingdom, and the fiscal year 2017/2018 for the United States. Because of rounding, the total figures may differ from the sum of their components.

Conventional signs:

e	estimated
-	nil
..	not available
|	break in continuity of series
.	decimal point

Nomenclature of NATO defence expenditure:

**1**	**Operating costs**
1.1	*Military personnel*
1.1.1	Pay and allowances
1.1.2	Employer's contributions to retirement funds
1.1.3	Other
1.2	*Civilian personnel*
1.2.1	Pay and allowances
1.2.2	Employer's contributions to retirement funds
1.3	*Pensions*
1.3.1	Paid to military retirees
1.3.2	Paid to civilian retirees
1.4	*Operations and maintenance*
1.4.1	Ammunition and explosives (excluding nuclear)
1.4.2	Petroleum products
1.4.3	Spare parts
1.4.4	Other equipment and supplies
1.4.5	Rents
1.4.6	Other operations and maintenance
**2**	**Procurement and construction**
2.1	*Major equipment*
2.1.1	Missile systems
2.1.2	Missiles (conventional weapons)
2.1.3	Nuclear weapons
2.1.4	Aircraft
2.1.5	Artillery
2.1.6	Combat vehicles
2.1.7	Engineering equipment
2.1.8	Weapons and small arms
2.1.9	Transport vehicles
2.1.10	Ships and harbour craft
2.1.11	Electronic and communications equipment
2.2	*National military construction*
2.3	NATO *common infrastructure*
2.3.1	Expenditure as host nation
2.3.2	Payments to other nations
2.3.3	Receipts from other nations
2.3.4	Land and utilities
**3**	**Research and development**
3.1	*Devoted to major equipment*
3.2	*Other*
**4**	**Other expenditure**
**5**	**Total**
6	Statistical discrepancy
**7**	**Adjusted total**

Main categories of defence expenditure:

- Equipment (Table 8a) – lines 2.1 + 3.1
- Personnel (Table 8a) – lines 1.1 + 1.2 + 1.3
- Infrastructure (Table 8b) – lines 2.2 + 2.3
- Other (Table 8b) – lines 1.4 + 3.2 + 4
