# Supplementary material for: A tale of five stories: Defence spending and economic growth in NATO´s countries
Source: PLoS One. 2021 Jan 11;16(1):e0245260. doi: 10.1371/journal.pone.0245260 (PMC7799761; doi:10.1371/journal.pone.0245260)
Supplement: S2 Fig — (PDF) [file pone.0245260.s002.pdf]

29 November/Novembre 2019

COMMUNIQUE
PR/CP(2019)123

Defence Expenditure of NATO Countries (2013-2019)

NATO collects defence expenditure data from Allies on a regular basis and presents aggregates and subsets of this information. Each Ally's Ministry of Defence reports current and estimated future defence expenditure according to an agreed definition of defence expenditure. The amounts represent payments by a national government actually made, or to be made, during the course of the fiscal year to meet the needs of its armed forces, those of Allies or of the Alliance. In the figures and tables that follow, NATO also uses economic and demographic information available from the Directorate-General for Economic and Financial Affairs of the European Commission (DG-ECFIN), and the Organisation for Economic Co-operation and Development (OECD).

In view of differences between both these sources and national GDP forecasts, and also the definition of NATO defence expenditure and national definitions, the figures shown in this report may diverge considerably from those which are quoted by media, published by national authorities or given in national budgets. Equipment expenditure includes expenditure on major equipment as well as on research and development devoted to major equipment. Personnel expenditure includes pensions paid to retirees.

The cut-off date for information used in this report was 21 November 2019. Figures for 2019 are estimates.

News and information is routinely placed on the NATO website. This includes audio files, transcripts and high resolution photographs, which are posted as soon as possible after events of media interest. Check the 'What's New' file.
Nouvelles et informations sont régulièrement affichées sur le site Web de l'OTAN, sous la forme de fichiers audio, de transcriptions et de photographies destinées à la publication. Elles sont disponibles le plus rapidement possible après les événements présentant un intérêt pour les médias. Cliquer sur "Quoi de neuf?"

North Atlantic Treaty Organisation – Organisation du Traité de l'Atlantique Nord
Press & Media – Presse & Médias
B-1110 Bruxelles Belgique
E-mail: press@hq.nato.int – Tel.: 32 2 707 50 41 – Fax: 32 2 707 13 99 – Internet: www.nato.int

Graph 1 : NATO Europe and Canada - defence expenditure

(annual real change, based on 2015 prices and exchange rates)

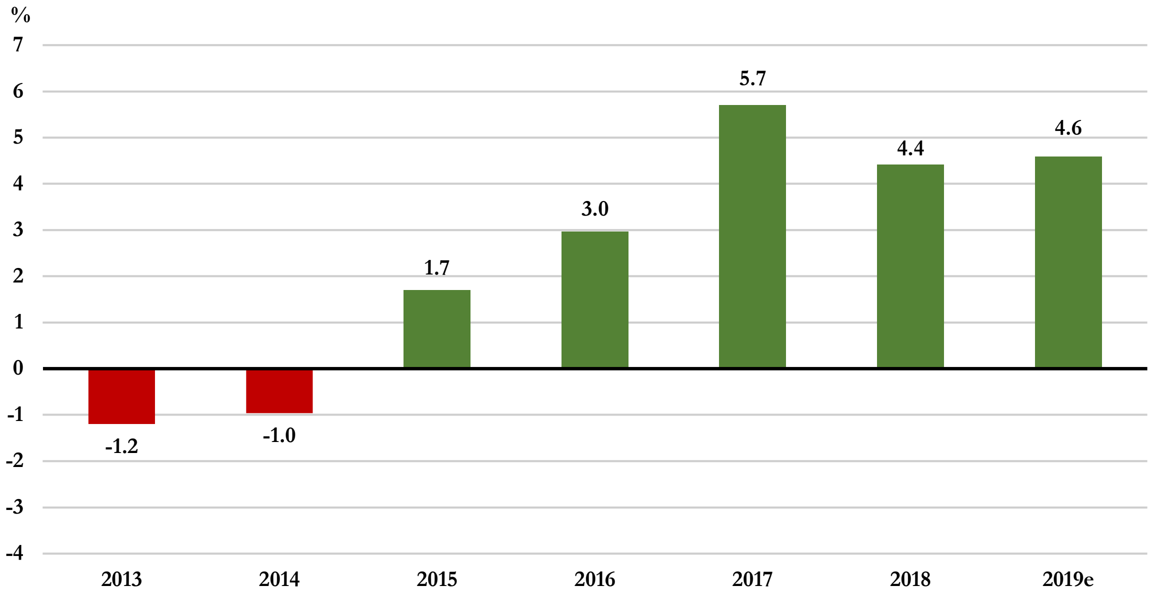

Notes: Figures for 2019 are estimates. The NATO Europe and Canada aggregate from 2017 onwards includes Montenegro, which became an Ally on 5 June 2017.

Graph 2 : Defence expenditure as a share of GDP and equipment expenditure as a share of defence expenditure 2019e

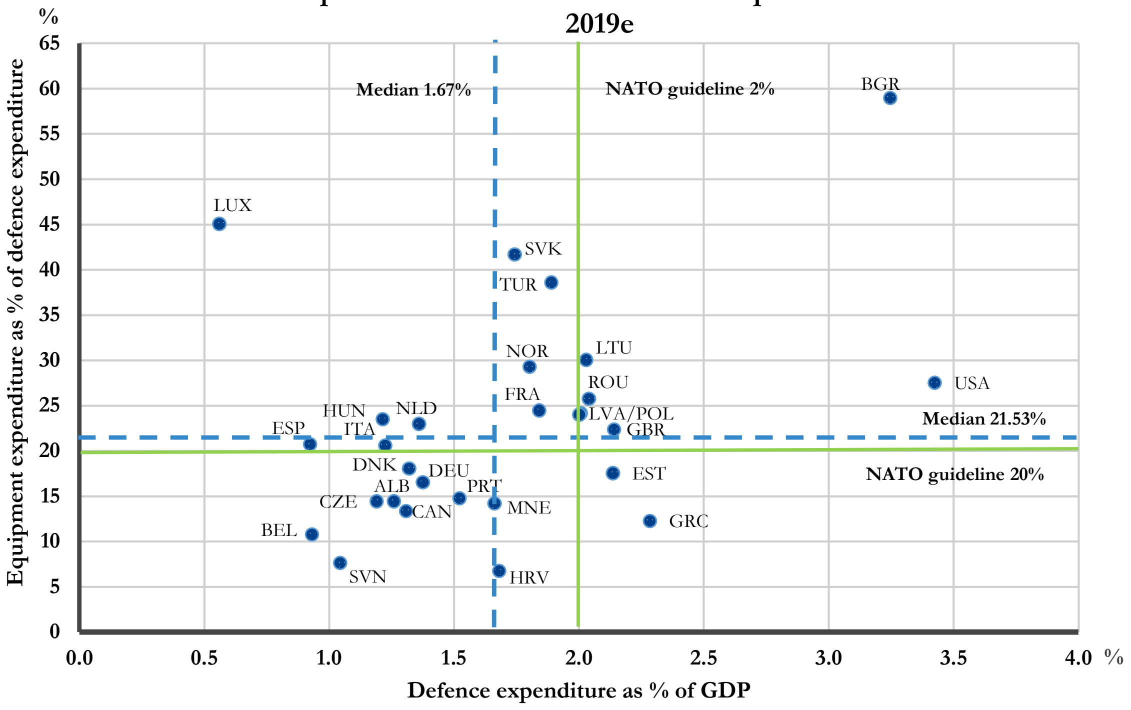

Notes: Figures for 2019 are estimates.

Graph 3 : Defence expenditure as a share of GDP (%)

(based on 2015 prices and exchange rates)

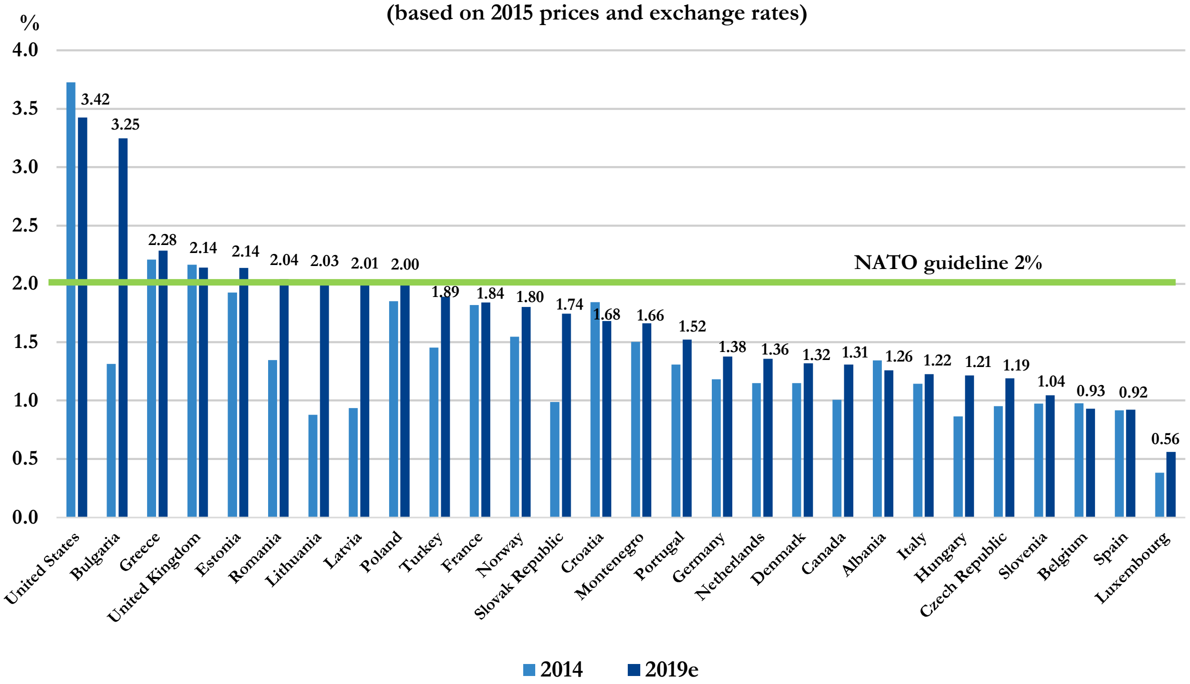

Notes: Figures for 2019 are estimates.

Graph 4 : Equipment expenditure as a share of defence expenditure (%)

(based on 2015 prices and exchange rates)

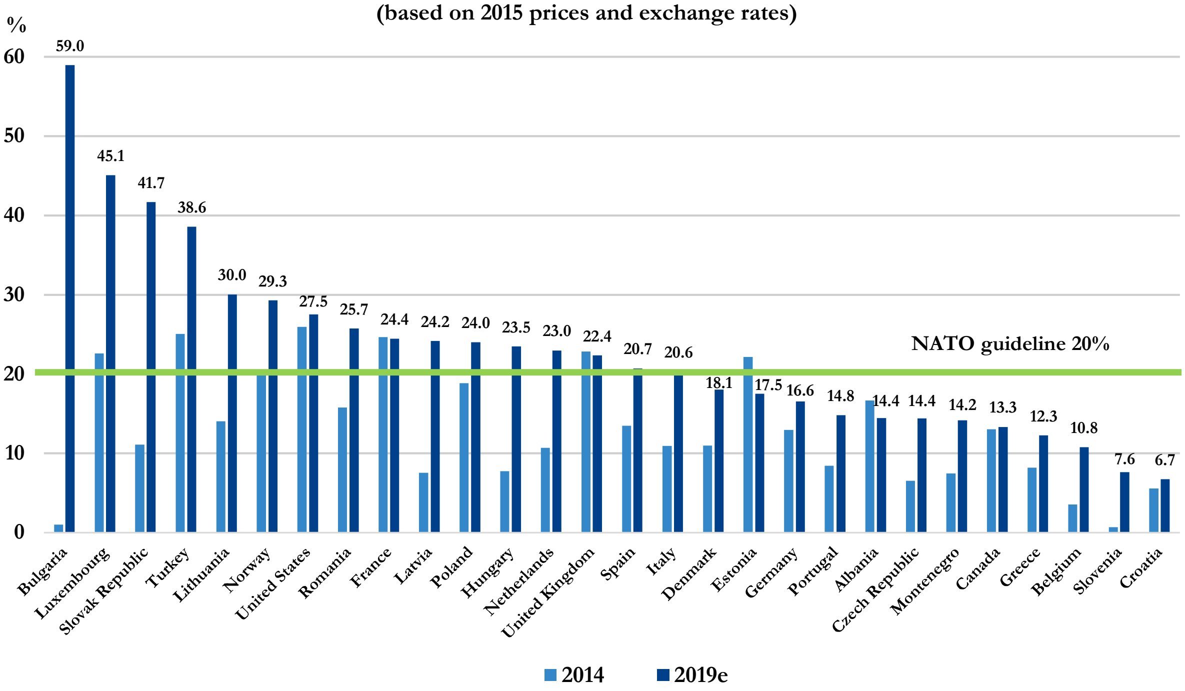

Notes: Figures for 2019 are estimates.

Graph 5 : Defence expenditure

(billion US dollars, based on 2015 prices and exchange rates)

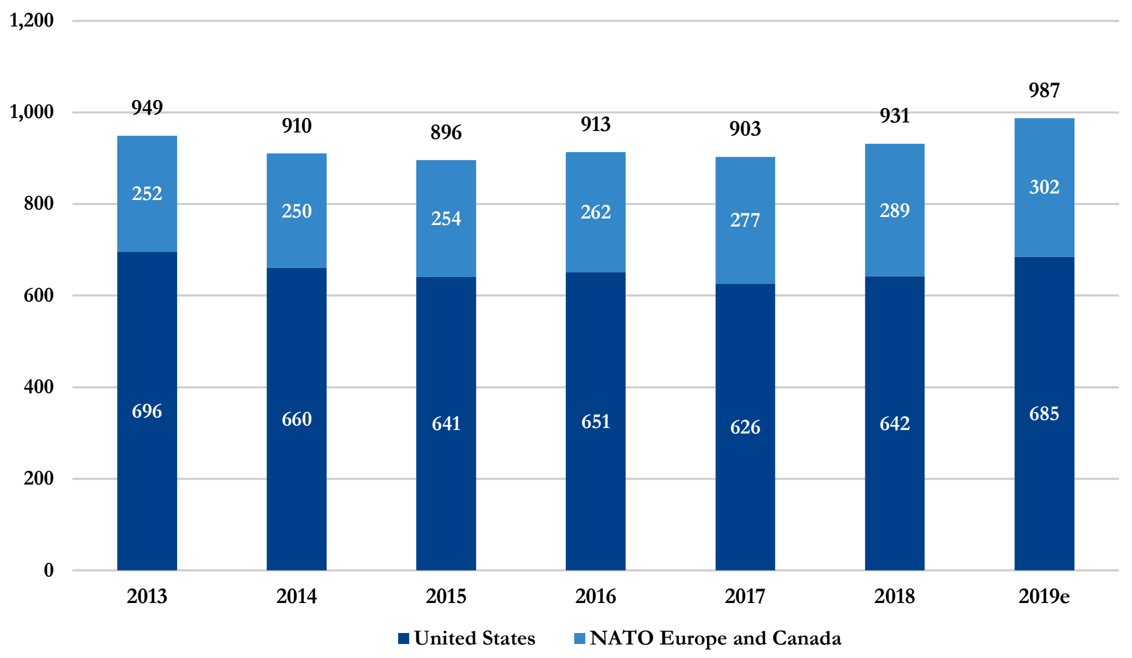

Notes: Figures for 2019 are estimates. The NATO Europe and Canada aggregate from 2017 onwards includes Montenegro, which became an Ally on 5 June 2017.

Graph 6 : NATO Europe and Canada - defence expenditure

(billion US dollars, based on 2015 prices and exchange rates)

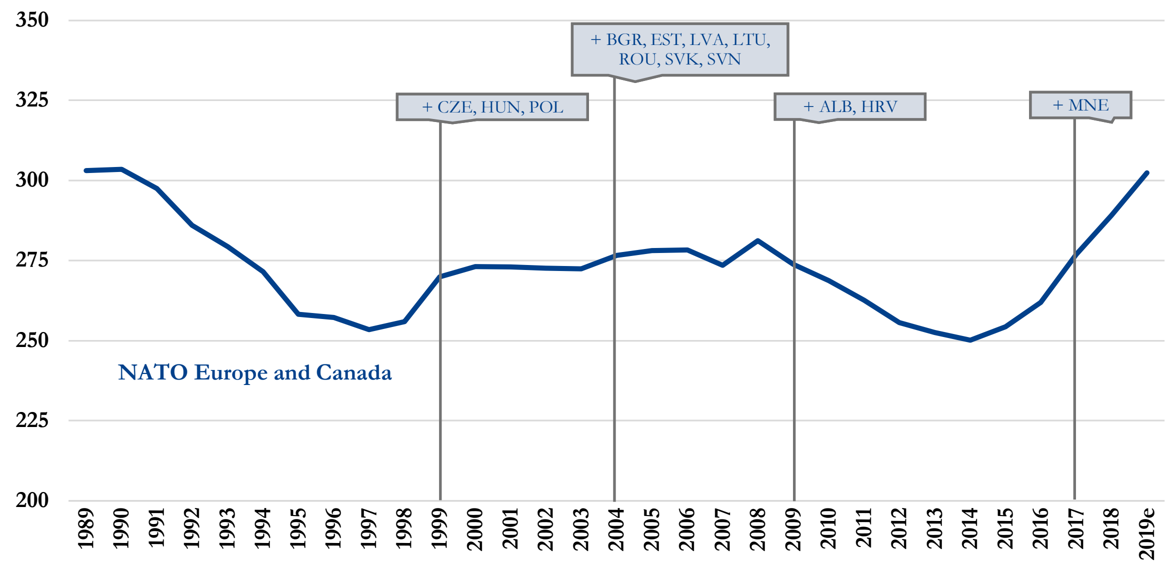

Notes: Figures for 2019 are estimates. Includes enlargements which took place in: 1999 (3 Allies), 2004 (7 Allies), 2009 (2 Allies) and 2017 (1 Ally).

Graph 7 : NATO Europe and Canada - cumulative defence spending over 2016

(billion US dollars, based on 2015 prices and exchange rates)

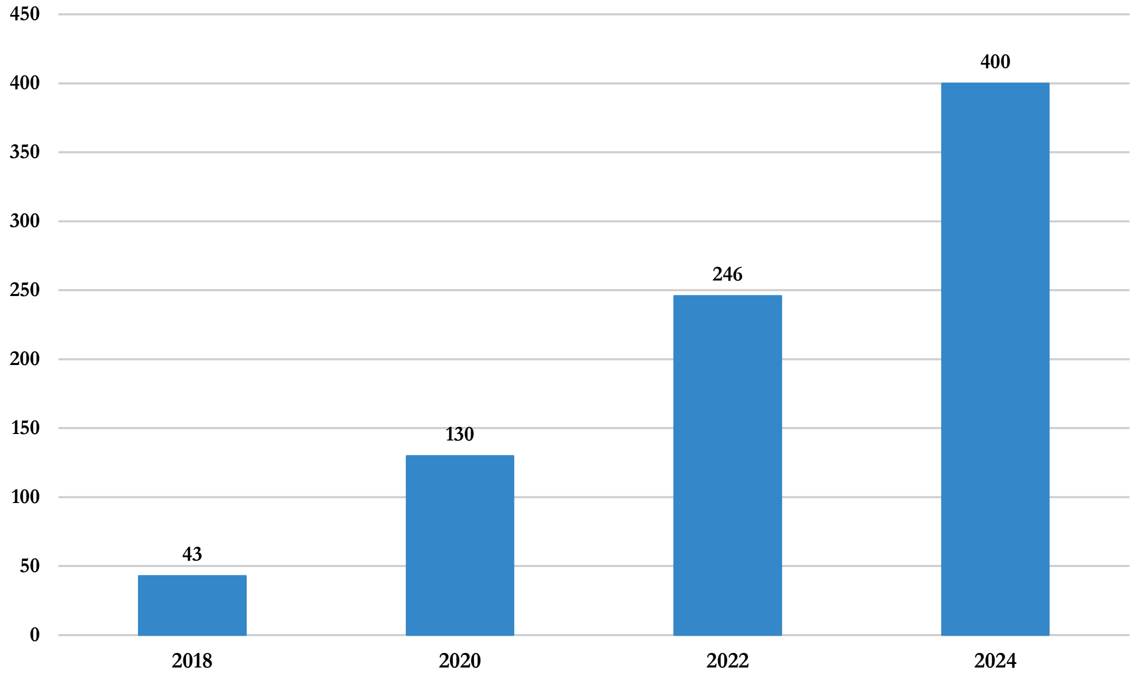

Figures from 2019 - 2024 are estimates.

Graph 8 : NATO Europe and Canada - major equipment expenditure

(annual real change, based on 2015 prices and exchange rates)

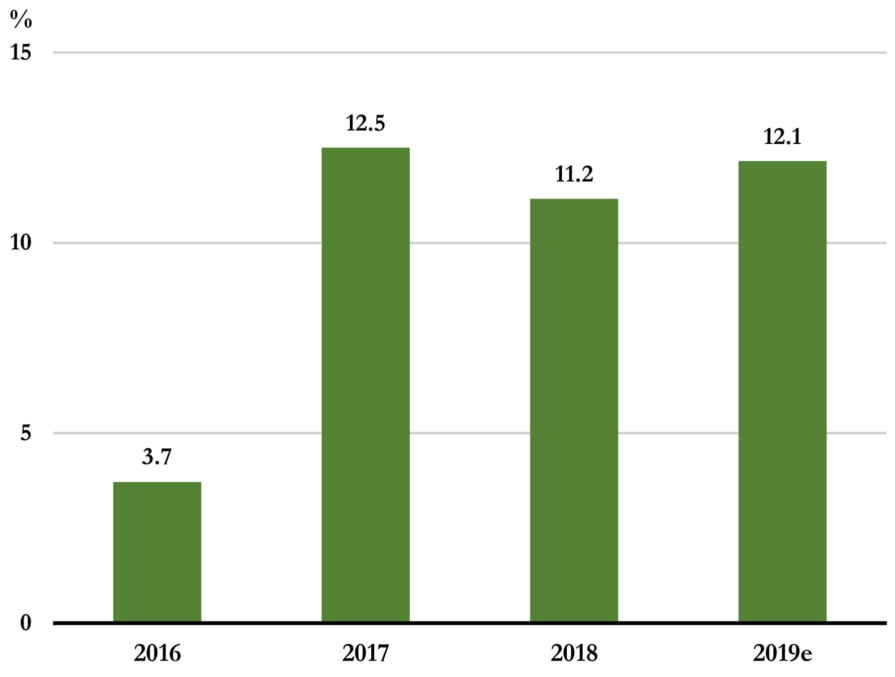

Notes: Figures for 2019 are estimates. The NATO Europe and Canada aggregate from 2017 onwards includes Montenegro, which became an Ally on 5 June 2017.

Table 1 : Defence expenditure

Million national currency units

	2013	2014	2015	2016	2017	2018	2019e
**Current prices**							
Albania (Leks)	19,022	18,788	16,671	16,250	17,199	18,995	21,702
Belgium (Euros)	3,964	3,913	3,789	3,848	3,932	4,101	4,303
Bulgaria (Leva)	1,196	1,102	1,116	1,186	1,255	1,593	3,716
Canada (Canadian dollars)	18,764	20,076	23,900	23,474	30,761	29,025	29,600
Croatia (Kunas)	4,848	6,113	6,057	5,696	6,120	6,068	6,683
Czech Republic (Koruny)	42,035	41,003	47,264	45,598	52,805	59,752	66,737
Denmark (Kroner)	23,682	22,769	22,633	24,190	24,961	28,787	30,389
Estonia (Euros)	361	386	418	450	479	514	586
France (Euros)	39,402	39,149	39,199	39,950	40,852	42,748	44,361
Germany (Euros)	34,593	34,749	35,898	37,598	40,265	42,127	47,878
Greece (Euros)	3,999	3,939	4,073	4,190	4,208	4,560	4,320
Hungary (Forint)	286,341	281,402	316,338	362,798	402,793	484,031	553,383
Italy (Euros)	20,078	18,427	17,642	20,226	21,166	21,183	21,408
Latvia* (Euros)	212	221	254	364	430	613	634
Lithuania* (Euros)	267	322	425	575	724	895	968
Luxembourg (Euros)	176	190	225	213	288	301	346
Montenegro (Euros)	49	52	51	56	58	64	81
Netherlands (Euros)	7,702	7,788	7,816	8,234	8,539	9,456	10,912
Norway (Kroner)	46,057	48,660	49,529	54,022	56,664	61,349	66,127
Poland* (Zlotys)	28,467	31,874	39,940	37,082	37,558	42,824	45,118
Portugal (Euros)	2,457	2,263	2,384	2,364	2,424	2,874	3,160
Romania* (New Lei)	8,160	9,014	10,337	10,738	14,765	17,181	20,932
Slovak Republic (Euros)	729	752	889	907	935	1,098	1,666
Slovenia (Euros)	382	366	361	406	422	463	508
Spain (Euros)	9,495	9,508	10,000	9,014	10,528	11,172	11,505
Turkey (Liras)	27,466	29,727	32,522	38,203	47,323	68,300	79,356
United Kingdom (Pounds)	39,824	39,902	38,940	41,590	43,257	45,206	46,861
United States (US dollars)	680,856	653,942	641,253	656,059	642,936	672,255	730,149
**Constant 2015 prices**							
Albania (Leks)	19,426	18,894	16,671	16,354	17,057	18,700	20,921
Belgium (Euros)	4,032	3,952	3,789	3,780	3,799	3,918	4,052
Bulgaria (Leva)	1,228	1,126	1,116	1,160	1,187	1,454	3,303
Canada (Canadian dollars)	18,956	19,894	23,900	23,292	29,769	27,623	27,962
Croatia (Kunas)	4,850	6,115	6,057	5,700	6,058	5,903	6,406
Czech Republic (Koruny)	43,580	41,481	47,264	45,029	51,408	56,954	61,781
Denmark (Kroner)	24,030	22,868	22,633	24,017	24,438	28,065	29,152
Estonia (Euros)	376	390	418	443	454	466	513
France (Euros)	40,081	39,595	39,199	39,873	40,506	41,993	43,074
Germany (Euros)	35,898	35,439	35,898	37,092	39,121	40,184	44,823
Greece (Euros)	3,908	3,925	4,073	4,195	4,192	4,522	4,259
Hungary (Forint)	302,053	286,921	316,338	359,267	385,004	442,784	485,889
Italy (Euros)	20,771	18,734	17,642	19,769	20,553	20,791	20,992
Latvia* (Euros)	216	221	254	361	413	565	561
Lithuania* (Euros)	271	323	425	567	685	819	863
Luxembourg (Euros)	180	190	225	211	280	281	314
Montenegro (Euros)	51	53	51	54	53	58	71
Netherlands (Euros)	7,780	7,796	7,816	8,162	8,340	9,041	10,292
Norway (Kroner)	44,905	47,287	49,529	54,643	55,176	56,588	59,853
Poland* (Zlotys)	28,829	32,119	39,940	36,967	36,723	41,353	42,635
Portugal (Euros)	2,525	2,309	2,384	2,323	2,347	2,743	2,982
Romania* (New Lei)	8,519	9,249	10,337	10,481	13,770	15,134	17,522
Slovak Republic (Euros)	727	750	889	911	928	1,068	1,581
Slovenia (Euros)	389	370	361	403	413	442	474
Spain (Euros)	9,525	9,557	10,000	8,988	10,369	10,896	11,123
Turkey (Liras)	31,814	32,054	32,522	35,341	39,495	49,074	48,959
United Kingdom (Pounds)	40,682	40,076	38,940	40,750	41,470	42,531	43,164
United States (US dollars)	696,291	660,062	641,253	651,201	626,380	642,012	685,099

Notes: Figures for 2019 are estimates.

* These Allies have national laws and political agreements which call for 2% of GDP to be spent on defence annually, consequently estimates are expected to change accordingly. For the past years, Allies' defence spending was based on the then available GDP data and Allies may, therefore, have met the 2% guideline when using those figures (In 2018, Lithuania met 2% using November 2018 OECD figures).

Table 2 : Defence expenditure

Million US dollars

	2013	2014	2015	2016	2017	2018	2019e
**Current prices and exchange rates**							
Albania	180	178	132	131	144	176	198
Belgium	5,264	5,192	4,202	4,256	4,431	4,840	4,921
Bulgaria	811	747	633	671	723	961	2,179
Canada	18,221	18,150	18,685	17,711	23,704	22,400	22,485
Croatia	850	1,064	883	837	924	966	1,032
Czech Republic	2,148	1,975	1,921	1,866	2,259	2,750	2,967
Denmark	4,217	4,057	3,364	3,593	3,780	4,559	4,651
Estonia	480	513	463	497	540	607	670
France	52,316	51,940	43,474	44,191	46,036	50,459	50,729
Germany	45,931	46,102	39,813	41,590	45,374	49,725	54,751
Greece	5,309	5,226	4,517	4,635	4,742	5,383	4,940
Hungary	1,280	1,210	1,132	1,289	1,468	1,791	2,080
Italy	26,658	24,448	19,566	22,373	23,852	25,004	24,482
Latvia*	281	293	281	403	484	724	724
Lithuania*	355	427	471	636	816	1,056	1,107
Luxembourg	234	253	249	236	325	355	395
Montenegro	65	69	57	62	65	76	92
Netherlands	10,226	10,332	8,668	9,108	9,622	11,162	12,478
Norway	7,839	7,722	6,142	6,431	6,850	7,544	7,708
Poland*	9,007	10,104	10,596	9,405	9,938	11,857	11,902
Portugal	3,262	3,003	2,644	2,615	2,732	3,392	3,613
Romania*	2,452	2,691	2,581	2,645	3,643	4,359	5,050
Slovak Republic	968	997	986	1,003	1,053	1,297	1,905
Slovenia	507	486	401	449	476	546	581
Spain	12,607	12,614	11,090	9,971	11,864	13,187	13,156
Turkey	14,427	13,583	11,957	12,649	12,972	14,145	13,919
United Kingdom	62,258	65,658	59,492	56,154	55,672	60,308	60,761
United States	680,856	653,942	641,253	656,059	642,936	672,255	730,149
**NATO Europe and Canada**	**288,088**	**288,965**	**254,347**	**255,345**	**274,490**	**299,631**	**309,479**
**NATO Total**	**968,944**	**942,907**	**895,600**	**911,404**	**917,426**	**971,886**	**1,039,628**
**Constant 2015 prices and exchange rates**							
Albania	154	150	132	130	135	148	166
Belgium	4,472	4,383	4,202	4,192	4,214	4,345	4,494
Bulgaria	696	638	633	657	673	824	1,872
Canada	14,820	15,553	18,685	18,210	23,273	21,596	21,860
Croatia	707	892	883	831	883	861	934
Czech Republic	1,772	1,686	1,921	1,831	2,090	2,315	2,512
Denmark	3,572	3,399	3,364	3,570	3,632	4,171	4,333
Estonia	417	432	463	491	504	517	569
France	44,453	43,914	43,474	44,222	44,923	46,573	47,771
Germany	39,813	39,304	39,813	41,138	43,387	44,567	49,712
Greece	4,335	4,353	4,517	4,652	4,650	5,015	4,723
Hungary	1,081	1,027	1,132	1,286	1,378	1,585	1,739
Italy	23,036	20,777	19,566	21,925	22,794	23,058	23,281
Latvia*	239	245	281	400	458	627	622
Lithuania*	301	358	471	629	759	908	957
Luxembourg	200	210	249	234	310	312	348
Montenegro	56	59	57	59	59	64	78
Netherlands	8,629	8,646	8,668	9,053	9,249	10,027	11,414
Norway	5,568	5,864	6,142	6,776	6,842	7,017	7,422
Poland*	7,648	8,521	10,596	9,807	9,742	10,970	11,311
Portugal	2,801	2,561	2,644	2,576	2,603	3,042	3,307
Romania*	2,127	2,309	2,581	2,617	3,438	3,778	4,374
Slovak Republic	806	832	986	1,011	1,029	1,184	1,754
Slovenia	431	411	401	447	457	490	525
Spain	10,564	10,599	11,090	9,968	11,500	12,084	12,336
Turkey	11,696	11,784	11,957	12,993	14,520	18,042	18,000
United Kingdom	62,153	61,227	59,492	62,257	63,357	64,978	65,944
United States	696,291	660,062	641,253	651,201	626,380	642,012	685,099
**NATO Europe and Canada**	**252,490**	**250,076**	**254,347**	**261,902**	**276,860**	**289,100**	**302,361**
**NATO Total**	**948,781**	**910,138**	**895,600**	**913,103**	**903,241**	**931,112**	**987,460**

Notes: Figures for 2019 are estimates. The NATO Europe and Canada and NATO Total aggregates from 2017 onwards include Montenegro, which became an Ally on 5 June 2017.

* These Allies have national laws and political agreements which call for 2% of GDP to be spent on defence annually, consequently estimates are expected to change accordingly. For the past years, Allies' defence spending was based on the then available GDP data and Allies may, therefore, have met the 2% guideline when using those figures (In 2018, Lithuania met 2% using November 2018 OECD figures).

Table 3 : Defence expenditure as a share of GDP and annual real change

Based on 2015 prices

	2013	2014	2015	2016	2017	2018	2019e
**Share of real GDP (%)**							
Albania	1.41	1.35	1.16	1.10	1.11	1.17	1.26
Belgium	1.01	0.98	0.92	0.91	0.90	0.91	0.93
Bulgaria	1.46	1.32	1.26	1.26	1.24	1.48	3.25
Canada	0.99	1.01	1.20	1.16	1.44	1.31	1.31
Croatia	1.46	1.84	1.78	1.62	1.67	1.59	1.68
Czech Republic	1.03	0.95	1.03	0.96	1.04	1.13	1.19
Denmark	1.23	1.15	1.11	1.15	1.15	1.30	1.32
Estonia	1.91	1.93	2.02	2.07	2.03	2.00	2.14
France	1.86	1.82	1.78	1.79	1.78	1.82	1.84
Germany	1.22	1.18	1.18	1.19	1.23	1.24	1.38
Greece	2.21	2.21	2.30	2.38	2.34	2.48	2.28
Hungary	0.95	0.86	0.92	1.02	1.05	1.15	1.21
Italy	1.27	1.14	1.07	1.18	1.21	1.21	1.22
Latvia*	0.93	0.94	1.04	1.45	1.59	2.08	2.01
Lithuania*	0.76	0.88	1.14	1.48	1.72	1.98	2.03
Luxembourg	0.38	0.38	0.44	0.40	0.52	0.51	0.56
Montenegro	1.47	1.50	1.40	1.42	1.35	1.39	1.66
Netherlands	1.17	1.15	1.13	1.16	1.15	1.21	1.36
Norway	1.50	1.55	1.59	1.73	1.71	1.73	1.80
Poland*	1.72	1.85	2.22	1.99	1.89	2.02	2.00
Portugal	1.44	1.31	1.33	1.27	1.25	1.43	1.52
Romania*	1.28	1.35	1.45	1.40	1.72	1.82	2.04
Slovak Republic	0.98	0.99	1.12	1.12	1.10	1.22	1.74
Slovenia	1.05	0.97	0.93	1.01	0.98	1.01	1.04
Spain	0.93	0.92	0.92	0.81	0.90	0.92	0.92
Turkey	1.52	1.45	1.39	1.46	1.52	1.85	1.89
United Kingdom	2.26	2.16	2.05	2.11	2.11	2.13	2.14
United States	4.03	3.73	3.52	3.52	3.31	3.30	3.42
**NATO Europe and Canada**	**1.47**	**1.43**	**1.43**	**1.44**	**1.48**	**1.52**	**1.57**
**NATO Total**	**2.76**	**2.59**	**2.48**	**2.49**	**2.40**	**2.42**	**2.52**
**Annual real change (%)**							
Albania	-4.30	-2.74	-11.76	-1.90	4.30	9.64	11.88
Belgium	-2.48	-1.99	-4.13	-0.25	0.52	3.12	3.43
Bulgaria*	9.53	-8.29	-0.84	3.88	2.33	22.52	127.17
Canada	-7.68	4.95	20.14	-2.54	27.81	-7.21	1.23
Croatia	-4.91	26.07	-0.94	-5.90	6.28	-2.55	8.52
Czech Republic	-3.13	-4.82	13.94	-4.73	14.17	10.79	8.48
Denmark	-8.37	-4.84	-1.03	6.12	1.75	14.84	3.87
Estonia	2.72	3.75	7.12	6.11	2.55	2.58	10.03
France	-0.02	-1.21	-1.00	1.72	1.59	3.67	2.57
Germany	-6.20	-1.28	1.30	3.33	5.47	2.72	11.54
Greece	-6.41	0.42	3.78	2.99	-0.06	7.87	-5.83
Hungary	-6.47	-5.01	10.25	13.57	7.16	15.01	9.74
Italy	-5.09	-9.81	-5.83	12.05	3.97	1.16	0.97
Latvia*	7.94	2.55	14.79	42.29	14.35	36.94	-0.74
Lithuania*	4.72	19.15	31.60	33.37	20.79	19.61	5.40
Luxembourg	3.70	5.33	18.59	-6.04	32.39	0.62	11.62
Montenegro	-8.44	4.49	-3.50	4.33	-0.77	8.39	22.74
Netherlands	-5.98	0.19	0.26	4.43	2.17	8.41	13.84
Norway	8.07	5.30	4.74	10.33	0.97	2.56	5.77
Poland*	0.07	11.41	24.35	-7.44	-0.66	12.61	3.10
Portugal	1.51	-8.56	3.24	-2.55	1.02	16.87	8.71
Romania*	8.39	8.57	11.76	1.39	31.38	9.91	15.78
Slovak Republic	-8.63	3.22	18.54	2.47	1.78	15.09	48.11
Slovenia	-11.19	-4.74	-2.39	11.55	2.32	7.15	7.20
Spain	-12.62	0.33	4.64	-10.12	15.37	5.08	2.09
Turkey	3.56	0.75	1.46	8.67	11.75	24.25	-0.24
United Kingdom	6.92	-1.49	-2.83	4.65	1.77	2.56	1.49
United States	-6.04	-5.20	-2.85	1.55	-3.81	2.50	6.71
**NATO Europe and Canada**	**-1.20**	**-0.96**	**1.71**	**2.97**	**5.71**	**4.42**	**4.59**
**NATO Total**	**-4.80**	**-4.07**	**-1.60**	**1.95**	**-1.08**	**3.09**	**6.05**

Notes: Figures for 2019 are estimates. The NATO Europe and Canada and NATO Total aggregates from 2017 onwards include Montenegro, which became an Ally on 5 June 2017.

* These Allies have national laws and political agreements which call for 2% of GDP to be spent on defence annually, consequently estimates are expected to change accordingly. For the past years, Allies' defence spending was based on the then available GDP data and Allies may, therefore, have met the 2% guideline when using those figures (In 2018, Lithuania met 2% using November 2018 OECD figures).

Table 4 : Defence expenditure real change 2014-2019

Million US dollars (2015 prices and exchange rates)

	2014	2019e	Real change 2014-2019e (%)	Share of real GDP 2014 (%)	Share of real GDP 2019e (%)
Albania	150	166	10.73	1.35	1.26
Belgium	4,383	4,494	2.53	0.98	0.93
Bulgaria	638	1,872	193.39	1.32	3.25
Canada	15,553	21,860	40.55	1.01	1.31
Croatia	892	934	4.77	1.84	1.68
Czech Republic	1,686	2,512	48.94	0.95	1.19
Denmark	3,399	4,333	27.48	1.15	1.32
Estonia	432	569	31.55	1.93	2.14
France	43,914	47,771	8.78	1.82	1.84
Germany	39,304	49,712	26.48	1.18	1.38
Greece	4,353	4,723	8.50	2.21	2.28
Hungary	1,027	1,739	69.35	0.86	1.21
Italy	20,777	23,281	12.05	1.14	1.22
Latvia*	245	622	153.86	0.94	2.01
Lithuania*	358	957	167.27	0.88	2.03
Luxembourg	210	348	65.68	0.38	0.56
Montenegro	59	78	32.92	1.50	1.66
Netherlands	8,646	11,414	32.02	1.15	1.36
Norway	5,864	7,422	26.57	1.55	1.80
Poland*	8,521	11,311	32.74	1.85	2.00
Portugal	2,561	3,307	29.12	1.31	1.52
Romania*	2,309	4,374	89.44	1.35	2.04
Slovak Republic	832	1,754	110.74	0.99	1.74
Slovenia	411	525	27.99	0.97	1.04
Spain	10,599	12,336	16.39	0.92	0.92
Turkey	11,784	18,000	52.74	1.45	1.89
United Kingdom	61,227	65,944	7.70	2.16	2.14
United States	660,062	685,099	3.79	3.73	3.42

Notes: Figures for 2019 are estimates.

* These Allies have national laws and political agreements which call for 2% of GDP to be spent on defence annually, consequently estimates are expected to change accordingly. For the past years, Allies' defence spending was based on the then available GDP data and Allies may, therefore, have met the 2% guideline when using those figures (In 2018, Lithuania met 2% using November 2018 OECD figures).

Table 5 : Real GDP

Billion US dollars (2015 prices and exchange rates)

	2013	2014	2015	2016	2017	2018	2019e
Albania	11	11	11	12	12	13	13
Belgium	443	448	456	463	471	477	483
Bulgaria	48	49	50	52	54	56	58
Canada	1,502	1,545	1,556	1,573	1,620	1,650	1,671
Croatia	48	48	50	51	53	54	56
Czech Republic	173	177	187	191	200	206	211
Denmark	291	296	303	310	317	321	328
Estonia	22	22	23	24	25	26	27
France	2,390	2,413	2,438	2,465	2,522	2,561	2,595
Germany	3,258	3,329	3,378	3,451	3,536	3,587	3,614
Greece	196	197	196	196	199	202	207
Hungary	114	119	123	126	131	138	143
Iceland	16	17	17	19	19	20	20
Italy	1,813	1,816	1,831	1,854	1,886	1,900	1,901
Latvia	26	26	27	28	29	30	31
Lithuania	39	41	42	42	44	46	47
Luxembourg	53	55	57	59	59	61	62
Montenegro	4	4	4	4	4	5	5
Netherlands	740	751	765	782	805	826	840
Norway	372	379	387	391	399	405	412
Poland	445	460	478	492	516	542	565
Portugal	194	196	199	203	209	213	217
Romania	166	171	178	186	199	208	214
Slovak Republic	82	84	88	91	93	97	101
Slovenia	41	42	43	44	47	49	50
Spain	1,141	1,157	1,199	1,237	1,274	1,307	1,336
Turkey	771	810	860	887	953	978	952
United Kingdom	2,749	2,830	2,896	2,948	3,002	3,044	3,081
United States	17,285	17,709	18,219	18,505	18,915	19,456	20,004
**NATO Europe and Canada**	**17,142**	**17,491**	**17,838**	**18,177**	**18,679**	**19,022**	**19,239**
**NATO Total**	**34,428**	**35,200**	**36,058**	**36,682**	**37,594**	**38,477**	**39,243**

Notes: Figures for 2019 are estimates. The NATO Europe and NATO Total aggregates from 2017 onwards include Montenegro, which became an Ally on 5 June 2017.

Table 6 : GDP per capita and defence expenditure per capita

2015 prices and exchange rates

	2013	2014	2015	2016	2017	2018	2019e
**GDP per capita (thousand US dollars)**							
Albania	3.8	3.9	4.0	4.1	4.3	4.4	4.6
Belgium	39.8	40.1	40.6	41.0	41.5	41.9	42.1
Bulgaria	6.6	6.7	7.0	7.3	7.7	7.9	8.2
Canada	42.7	43.5	43.4	43.4	44.1	44.5	44.7
Croatia	11.4	11.4	11.8	12.3	12.8	13.2	13.6
Czech Republic	16.4	16.9	17.7	18.1	18.9	19.3	19.8
Denmark	51.8	52.4	53.3	54.1	55.0	55.5	56.2
Estonia	16.5	17.1	17.4	18.0	18.9	19.6	20.1
France	36.2	36.4	36.6	36.9	37.7	38.2	38.6
Germany	40.4	41.1	41.4	41.9	42.8	43.3	43.4
Greece	17.8	18.1	18.1	18.2	18.5	18.9	19.3
Hungary	11.5	12.1	12.5	12.8	13.4	14.1	14.7
Iceland	50.2	50.7	52.6	55.3	56.5	58.6	58.7
Italy	29.9	29.9	30.1	30.6	31.2	31.4	31.4
Latvia	12.8	13.1	13.6	14.1	14.8	15.7	16.2
Lithuania	13.3	13.9	14.3	14.8	15.7	16.4	17.0
Luxembourg	96.7	98.5	100.4	100.2	99.6	99.9	99.7
Montenegro	6.2	6.3	6.5	6.7	7.1	7.4	7.6
Netherlands	44.0	44.5	45.2	45.9	47.0	47.9	48.4
Norway	73.2	73.8	74.5	74.7	75.6	76.1	76.8
Poland	11.6	12.0	12.4	12.8	13.4	14.1	14.7
Portugal	18.6	18.8	19.3	19.7	20.3	20.8	21.2
Romania	8.3	8.6	9.0	9.5	10.2	10.6	11.0
Slovak Republic	15.1	15.5	16.2	16.7	17.2	17.9	18.4
Slovenia	19.9	20.4	20.9	21.5	22.6	23.5	24.3
Spain	24.5	24.9	25.8	26.6	27.4	28.0	28.5
Turkey	10.2	10.6	11.1	11.3	12.1	12.2	11.7
United Kingdom	42.9	43.8	44.5	44.9	45.5	45.8	46.0
United States	54.6	55.5	56.7	57.2	58.0	59.3	60.5
**NATO Europe and Canada**	**28.7**	**29.2**	**29.7**	**30.1**	**30.8**	**31.3**	**31.5**
**NATO Total**	**37.7**	**38.3**	**39.1**	**39.5**	**40.3**	**41.1**	**41.7**
**Defence expenditure per capita (US dollars)**							
Albania	53	52	46	45	47	52	58
Belgium	402	392	374	371	371	381	392
Bulgaria	96	88	88	92	95	117	268
Canada	422	438	521	502	634	583	585
Croatia	166	210	210	199	214	210	229
Czech Republic	169	160	182	173	197	218	236
Denmark	636	602	592	623	630	720	742
Estonia	316	329	353	373	383	392	430
France	674	662	653	663	672	695	710
Germany	494	485	487	500	525	538	597
Greece	395	400	417	432	432	467	441
Hungary	109	104	115	131	141	162	178
Italy	380	342	322	362	377	381	385
Latvia	119	123	142	204	236	326	325
Lithuania	102	122	162	219	269	324	346
Luxembourg	366	377	438	401	520	511	558
Montenegro	91	95	91	95	95	103	127
Netherlands	514	513	512	532	540	582	658
Norway	1,096	1,141	1,183	1,294	1,297	1,321	1,384
Poland	199	221	276	255	254	286	295
Portugal	268	246	255	250	253	296	323
Romania	106	116	130	133	175	193	225
Slovak Republic	149	154	182	186	189	217	322
Slovenia	209	199	194	217	221	237	253
Spain	227	228	239	215	247	258	264
Turkey	154	154	154	166	184	225	222
United Kingdom	970	948	914	948	959	978	985
United States	2,199	2,069	1,996	2,012	1,922	1,956	2,072
**NATO Europe and Canada**	**2,022**	**1,906**	**1,848**	**1,860**	**1,791**	**1,816**	**1,921**
**NATO Total**	**1,039**	**991**	**971**	**984**	**969**	**993**	**1,048**

Notes: Figures for 2019 are estimates. The NATO Europe and NATO Total aggregates from 2017 onwards include Montenegro, which became an Ally on 5 June 2017.

Table 7 : Military personnel

Thousands

	2013	2014	2015	2016	2017	2018	2019e
Albania	6.8	6.7	6.2	5.8	6.8	6.8	6.8
Belgium	30.7	30.5	29.7	28.8	27.8	26.5	25.9
Bulgaria	28.0	27.5	24.9	24.7	24.3	24.4	24.8
Canada	67.5	65.9	70.3	70.5	68.2	70.3	69.4
Croatia	15.8	15.4	15.1	14.8	14.8	15.0	15.1
Czech Republic	19.8	20.2	21.5	22.7	23.8	24.7	25.0
Denmark	18.1	16.9	17.2	17.3	16.7	17.2	18.0
Estonia	6.3	6.3	6.0	6.1	6.0	6.2	6.3
France	213.1	207.0	204.8	208.1	208.2	208.2	207.8
Germany	184.0	178.8	177.2	177.9	179.8	181.5	182.0
Greece	110.4	107.3	104.4	106.0	104.6	106.8	107.6
Hungary	18.1	17.5	17.4	17.9	17.8	18.7	19.7
Italy	188.9	183.5	178.4	176.3	174.6	174.1	178.1
Latvia	4.6	4.6	4.8	5.2	5.5	5.9	6.4
Lithuania	8.4	8.6	11.8	11.8	13.5	14.3	15.7
Luxembourg	0.9	0.8	0.8	0.8	0.8	0.9	0.9
Montenegro	1.9	1.9	1.7	1.5	1.5	1.5	1.5
Netherlands	42.2	41.2	40.6	40.0	39.5	39.3	41.7
Norway	19.9	21.0	20.9	20.5	20.2	20.2	20.7
Poland	100.0	99.0	98.9	101.6	105.3	109.5	118.5
Portugal	32.7	30.7	28.3	29.8	27.8	26.9	28.6
Romania	66.2	65.1	64.5	63.4	61.6	61.5	73.1
Slovak Republic	12.3	12.4	12.4	12.2	11.9	11.7	11.7
Slovenia	6.9	6.8	6.6	6.5	6.3	6.2	6.3
Spain	121.8	121.8	121.6	121.0	117.7	117.4	121.0
Turkey	426.6	426.6	384.8	359.3	416.7	444.3	435.5
United Kingdom	179.4	168.7	141.4	139.5	156.7	157.1	153.3
United States	1,382.3	1,338.2	1,314.1	1,301.4	1,307.5	1,322.5	1,338.1
**NATO Europe and Canada**	**1,930**	**1,891**	**1,811**	**1,788**	**1,859**	**1,897**	**1,921**
**NATO Total**	**3,312**	**3,229**	**3,125**	**3,090**	**3,166**	**3,220**	**3,259**

Notes: Figures for 2019 are estimates. The NATO Europe and NATO Total aggregates from 2017 onwards include Montenegro, which became an Ally on 5 June 2017.

Table 8a : Distribution of defence expenditure by main category

Percentage of total defence expenditure

	2013	2014	2015	2016	2017	2018	2019e
**Equipment (a)**							
Albania	16.29	16.65	8.92	8.01	6.96	9.42	14.42
Belgium	2.84	3.52	3.44	4.72	6.52	10.15	10.78
Bulgaria	4.52	1.03	3.47	9.15	8.10	9.65	58.97
Canada	11.16	13.03	10.47	10.61	10.70	11.94	13.34
Croatia	10.72	5.56	8.01	7.51	5.69	3.37	6.73
Czech Republic	9.49	6.53	11.75	6.70	11.55	11.16	14.42
Denmark	11.26	10.99	11.50	13.68	10.39	11.66	18.06
Estonia	14.48	22.15	12.82	17.86	19.22	16.73	17.54
France	28.56	24.64	25.04	24.44	24.17	23.66	24.45
Germany	12.74	12.94	11.93	12.21	11.77	12.36	16.55
Greece	12.06	8.17	10.40	13.45	11.28	11.03	12.27
Hungary	11.08	7.76	9.75	13.37	15.34	20.35	23.48
Italy	12.51	10.92	9.72	19.09	20.68	21.12	20.57
Latvia	12.09	7.55	13.60	19.05	15.01	31.19	24.18
Lithuania	9.23	14.06	21.55	30.06	31.61	36.98	30.04
Luxembourg	14.57	22.61	33.33	30.07	42.06	45.18	45.06
Montenegro	1.32	7.46	5.43	4.46	4.95	11.05	14.19
Netherlands	12.57	10.68	11.16	14.14	14.75	16.39	22.98
Norway	18.89	20.42	21.83	23.37	24.63	25.60	29.27
Poland	13.90	18.84	33.20	21.62	22.04	27.51	24.01
Portugal	8.65	8.43	8.70	9.95	11.42	9.78	14.78
Romania	10.71	15.77	19.65	20.43	33.34	33.48	25.74
Slovak Republic	7.39	11.12	18.28	15.32	17.74	22.27	41.68
Slovenia	1.27	0.66	1.85	1.02	4.04	5.86	7.63
Spain	12.37	13.49	14.82	6.65	20.39	21.83	20.72
Turkey	26.89	25.08	25.13	25.55	30.30	37.64	38.60
United Kingdom	21.89	22.82	21.75	21.24	22.29	22.19	22.35
United States	25.83	25.97	25.41	25.05	25.73	27.06	27.51
**Personnel (b)**							
Albania	75.25	68.05	78.15	68.05	68.20	70.70	64.18
Belgium	77.34	77.84	78.23	76.80	75.20	70.69	68.65
Bulgaria	65.37	72.84	73.66	65.64	68.33	62.99	28.73
Canada	52.44	50.90	53.76	53.11	57.37	51.02	49.40
Croatia	68.06	76.55	72.28	75.40	71.72	76.96	72.96
Czech Republic	62.03	61.40	55.27	61.97	56.11	54.57	53.16
Denmark	51.74	51.27	52.01	49.51	47.01	49.88	48.19
Estonia	39.83	38.62	39.56	38.70	34.89	34.27	35.47
France	49.23	48.59	47.79	47.94	47.98	46.90	45.78
Germany	49.86	50.67	49.86	48.35	48.96	47.99	44.02
Greece	74.56	77.18	72.05	73.13	76.56	78.76	76.82
Hungary	48.96	49.77	48.21	49.66	42.41	39.98	41.72
Italy	75.00	76.41	77.55	70.79	67.58	65.66	..
Latvia	52.98	52.97	50.06	43.87	38.59	34.21	34.91
Lithuania	66.53	57.53	48.49	45.50	40.79	37.47	42.36
Luxembourg	51.10	49.31	42.77	45.56	34.40	33.42	30.25
Montenegro	87.68	78.53	78.03	75.32	80.44	72.87	64.17
Netherlands	58.53	56.50	55.51	51.77	52.19	51.16	45.25
Norway	42.21	40.64	39.96	38.60	37.08	36.43	34.23
Poland	57.70	51.45	41.96	47.15	50.04	46.14	48.47
Portugal	79.85	81.27	81.90	81.38	80.19	74.84	69.77
Romania	78.99	71.15	63.30	65.01	54.67	54.48	54.79
Slovak Republic	70.14	69.14	56.24	58.72	58.21	54.74	40.61
Slovenia	80.52	82.31	82.23	76.03	75.04	72.38	68.65
Spain	68.25	67.34	65.18	72.61	61.64	59.64	60.80
Turkey	54.58	56.88	56.82	57.60	51.02	45.18	46.75
United Kingdom	37.85	36.59	36.80	35.27	34.54	33.82	34.52
United States	34.38	35.45	36.64	45.01	41.22	39.28	38.62

Notes: Figures for 2019 are estimates.
(a) Equipment expenditure includes major equipment expenditure and R&D devoted to major equipment.
(b) Personnel expenditure includes military and civilian expenditure and pensions.

Table 8b : Distribution of defence expenditure by main category

Percentage of total defence expenditure

	2013	2014	2015	2016	2017	2018	2019e
**Infrastructure (c)**							
Albania	1.17	0.86	1.40	1.37	0.92	1.09	1.98
Belgium	2.28	1.81	0.93	0.96	1.05	1.43	1.30
Bulgaria	0.47	0.63	1.27	0.63	0.83	2.62	3.00
Canada	4.12	3.81	3.63	3.03	2.98	3.58	3.45
Croatia	1.21	1.24	1.98	1.26	3.59	1.00	1.64
Czech Republic	2.72	2.34	3.32	3.91	3.99	5.31	7.04
Denmark	1.16	0.97	1.09	2.16	1.95	1.49	1.74
Estonia	11.54	8.20	8.45	12.15	11.27	8.63	5.50
France	2.30	2.33	2.80	2.70	2.88	3.51	3.12
Germany	3.55	3.75	3.60	3.39	4.06	4.15	3.82
Greece	0.63	1.10	0.65	0.58	0.79	0.62	1.25
Hungary	2.32	1.07	1.21	1.13	1.64	4.85	3.52
Italy	1.57	1.40	1.30	0.70	0.94	1.92	..
Latvia	6.26	8.89	6.64	12.83	15.02	6.97	9.27
Lithuania	2.04	2.17	2.16	3.59	3.92	2.24	5.02
Luxembourg	11.81	10.26	7.79	6.64	4.64	5.05	6.71
Montenegro	0.09	0.96	2.47	2.41	0.88	2.24	3.97
Netherlands	2.74	4.77	3.19	3.90	3.02	3.46	3.19
Norway	5.33	5.71	5.30	6.56	6.93	6.67	6.44
Poland	5.62	5.47	4.74	4.62	4.21	3.45	5.35
Portugal	0.04	0.11	0.25	0.06	0.03	0.12	0.32
Romania	1.16	1.09	1.27	2.77	2.09	1.54	4.42
Slovak Republic	0.29	0.57	1.99	3.75	2.97	2.00	1.90
Slovenia	1.33	0.65	0.61	1.14	0.45	1.40	0.65
Spain	0.67	0.66	0.97	0.97	0.68	0.64	1.10
Turkey	2.72	2.77	2.56	2.42	2.95	2.53	2.04
United Kingdom	2.04	1.95	1.63	1.87	2.25	2.99	2.17
United States	2.08	1.71	1.45	1.22	1.23	1.17	1.27
**Other (d)**							
Albania	7.30	14.44	11.53	22.57	23.92	18.79	19.41
Belgium	17.53	16.83	17.40	17.52	17.23	17.72	19.27
Bulgaria	29.64	25.51	21.60	24.57	22.74	24.74	9.30
Canada	32.28	32.26	32.14	33.25	28.95	33.46	33.81
Croatia	20.01	16.65	17.73	15.83	18.99	18.67	18.67
Czech Republic	25.75	29.73	29.65	27.43	28.35	28.95	25.39
Denmark	35.84	36.78	35.40	34.65	40.66	36.97	32.01
Estonia	34.14	31.03	39.18	31.30	34.62	40.36	41.49
France	19.91	24.43	24.37	24.92	24.97	25.92	26.65
Germany	33.84	32.63	34.61	36.05	35.20	35.49	35.61
Greece	12.75	13.55	16.90	12.84	11.37	9.60	9.66
Hungary	37.64	41.40	40.83	35.84	40.62	34.82	31.29
Italy	10.93	11.27	11.42	9.42	10.80	11.30	..
Latvia	28.68	30.59	29.69	24.25	31.38	27.63	31.64
Lithuania	22.20	26.24	27.79	20.85	23.67	23.30	22.59
Luxembourg	22.52	17.82	16.11	17.73	18.90	16.35	17.98
Montenegro	10.91	13.06	14.07	17.80	13.73	13.84	17.67
Netherlands	26.16	28.05	30.14	30.20	30.04	28.99	28.58
Norway	33.88	33.24	32.90	31.46	31.36	31.30	30.06
Poland	22.78	24.24	20.11	26.61	23.71	22.89	22.17
Portugal	11.46	10.19	9.15	8.61	8.35	15.26	15.13
Romania	9.13	11.98	15.78	11.79	9.90	10.50	15.06
Slovak Republic	22.19	19.16	23.49	22.22	21.08	20.99	15.80
Slovenia	16.88	16.38	15.31	21.80	20.47	20.36	23.07
Spain	18.71	18.50	19.03	19.78	17.28	17.89	17.39
Turkey	15.80	15.27	15.49	14.43	15.73	14.65	12.61
United Kingdom	38.22	38.63	39.82	41.62	40.92	41.00	40.95
United States	37.72	36.87	36.51	28.73	31.83	32.49	32.59

Notes: Figures for 2019 are estimates.

(c) Infrastructure expenditure includes NATO common infrastructure and national military construction.

(d) Other expenditure includes operations and maintenance expenditure, other R&D expenditure and expenditure not allocated among above-mentioned categories.

NATO defence expenditure

NATO defines defence expenditure as payments made by a national government specifically to meet the needs of its armed forces, those of Allies or of the Alliance. A major component of defence expenditure is payments for Armed Forces financed from within the Ministry of Defence (MoD) budget. Armed Forces include Land, Maritime and Air forces as well as Joint formations such as Administration and Command, Special Operations Forces, Medical Service, Logistic Command etc. They might also include "Other Forces" like Ministry of Interior troops, national police forces, gendarmerie, carabinieri, coast guards etc. In such cases, expenditure should be included only in proportion to the forces that are trained in military tactics, are equipped as a military force, can operate under direct military authority in deployed operations, and can, realistically, be deployed outside national territory in support of a military force. Also, expenditure on Other Forces financed through the budgets of ministries other than MoD should be included in defence expenditure.

Pension payments made directly by the government to retired military and civilian employees of military departments should be included regardless of whether these payments are made from the budget of the MoD or other ministries.

Expenditure for peacekeeping and humanitarian operations (paid by MoD or other ministries), the destruction of weapons, equipment and ammunition, contributions to eligible NATO-managed trust funds, and the costs associated with inspection and control of equipment destruction are included in defence expenditure.

Research and development (R&D) costs are to be included in defence expenditure. R&D costs should also include expenditure for those projects that do not successfully lead to production of equipment.

Expenditure for the military component of mixed civilian-military activities is included, but only when the military component can be specifically accounted for or estimated.

Expenditure on NATO common infrastructure is included in the total defence expenditure of each Ally only to the extent of that nation's net contribution.

War damage payments and spending on civil defence are both excluded from the NATO definition of defence expenditure.

NATO uses United States dollars (USD) as the common currency denominator. The exchange rate applied to each Ally is the average annual rate published by the International Monetary Fund (IMF).

Note to readers:

Iceland has no armed forces. For nations of the Euro zone, and Montenegro, monetary values in national currency are expressed in Euros for all years. Estonia adopted Euros from 2011, Latvia from 2014, and Lithuania from 2015. Montenegro joined the Alliance in 2017.

To avoid any ambiguity, the fiscal year has been designated by the year which includes the highest number of months: e.g. 2019 represents the fiscal year 2019/2020 for Canada and United Kingdom, and the fiscal year 2018/2019 for the United States. Because of rounding, the total figures may differ from the sum of their components.

Conventional signs:

e	estimated
-	nil
..	not available
|	break in continuity of series
.	decimal point

Nomenclature of NATO defence expenditure:

**1**	**Operating costs**
1.1	*Military personnel*
1.1.1	Pay and allowances
1.1.2	Employer's contributions to retirement funds
1.1.3	Other
1.2	*Civilian personnel*
1.2.1	Pay and allowances
1.2.2	Employer's contributions to retirement funds
1.3	*Pensions*
1.3.1	Paid to military retirees
1.3.2	Paid to civilian retirees
1.4	*Operations and maintenance*
1.4.1	Ammunition and explosives (excluding nuclear)
1.4.2	Petroleum products
1.4.3	Spare parts
1.4.4	Other equipment and supplies
1.4.5	Rents
1.4.6	Other operations and maintenance
**2**	**Procurement and construction**
2.1	*Major equipment*
2.1.1	Missile systems
2.1.2	Missiles (conventional weapons)
2.1.3	Nuclear weapons
2.1.4	Aircraft
2.1.5	Artillery
2.1.6	Combat vehicles
2.1.7	Engineering equipment
2.1.8	Weapons and small arms
2.1.9	Transport vehicles
2.1.10	Ships and harbour craft
2.1.11	Electronic and communications equipment
2.2	*National military construction*
2.3	NATO *common infrastructure*
2.3.1	Expenditure as host nation
2.3.2	Payments to other nations
2.3.3	Receipts from other nations
2.3.4	Land and utilities
**3**	**Research and development**
3.1	*Devoted to major equipment*
3.2	*Other*
**4**	**Other expenditure**
**5**	**Total**
6	Statistical discrepancy
**7**	**Adjusted total**

Main categories of defence expenditure:

- Equipment (Table 8a) – lines 2.1 + 3.1
- Personnel (Table 8a) – lines 1.1 + 1.2 + 1.3
- Infrastructure (Table 8b) – lines 2.2 + 2.3
- Other (Table 8b) – lines 1.4 + 3.2 + 4
